# UAV-Based Thermal Imaging for High-Throughput Field Phenotyping of Black Poplar Response to Drought

**DOI:** 10.3389/fpls.2017.01681

**Published:** 2017-09-27

**Authors:** Riccardo Ludovisi, Flavia Tauro, Riccardo Salvati, Sacha Khoury, Giuseppe Mugnozza Scarascia, Antoine Harfouche

**Affiliations:** ^1^Department for Innovation in Biological, Agro-food and Forest Systems, University of Tuscia, Viterbo, Italy; ^2^Department of Plant Sciences, University of Cambridge, Cambridge, United Kingdom

**Keywords:** UAV remote sensing, high-throughput field phenotyping (HTFP), phenomics, poplar thermal imagery, image processing, stomatal conductance, drought

## Abstract

Poplars are fast-growing, high-yielding forest tree species, whose cultivation as second-generation biofuel crops is of increasing interest and can efficiently meet emission reduction goals. Yet, breeding elite poplar trees for drought resistance remains a major challenge. Worldwide breeding programs are largely focused on intra/interspecific hybridization, whereby *Populus nigra* L. is a fundamental parental pool. While high-throughput genotyping has resulted in unprecedented capabilities to rapidly decode complex genetic architecture of plant stress resistance, linking genomics to phenomics is hindered by technically challenging phenotyping. Relying on unmanned aerial vehicle (UAV)-based remote sensing and imaging techniques, high-throughput field phenotyping (HTFP) aims at enabling highly precise and efficient, non-destructive screening of genotype performance in large populations. To efficiently support forest-tree breeding programs, ground-truthing observations should be complemented with standardized HTFP. In this study, we develop a high-resolution (leaf level) HTFP approach to investigate the response to drought of a full-sib F_2_ partially inbred population (termed here ‘POP6’), whose F_1_ was obtained from an intraspecific *P. nigra* controlled cross between genotypes with highly divergent phenotypes. We assessed the effects of two water treatments (well-watered and moderate drought) on a population of 4603 trees (503 genotypes) hosted in two adjacent experimental plots (1.67 ha) by conducting low-elevation (25 m) flights with an aerial drone and capturing 7836 thermal infrared (TIR) images. TIR images were undistorted, georeferenced, and orthorectified to obtain radiometric mosaics. Canopy temperature (*T*_c_) was extracted using two independent semi-automated segmentation techniques, eCognition- and Matlab-based, to avoid the mixed-pixel problem. Overall, results showed that the UAV platform-based thermal imaging enables to effectively assess genotype variability under drought stress conditions. *T*_c_ derived from aerial thermal imagery presented a good correlation with ground-truth stomatal conductance (*g*_s_) in both segmentation techniques. Interestingly, the HTFP approach was instrumental to detect drought-tolerant response in 25% of the population. This study shows the potential of UAV-based thermal imaging for field phenomics of poplar and other tree species. This is anticipated to have tremendous implications for accelerating forest tree genetic improvement against abiotic stress.

## Introduction

Fast growing *Populus* clones are among the most common lignocellulosic feedstocks for second-generation bioenergy production in Europe (Amichev et al., 2010; Sannigrahi et al., 2010; Sabatti et al., 2014; Djomo et al., 2015). A recent report of the International Poplar Commission indicates that the total area of short rotation coppice (SRC) *Populus* across Europe is about 23,502 ha (FAO, 2016). However, the current surface area planted with SRC *Populus* in Europe was estimated at about 45,000 ha by Alasia Franco Vivai (Franco Alasia, Personal Communication), taking into account the establishment of few thousands ha in recent years in Poland. Compared to alternative bioenergy crops, SRC plantations offer versatile year-round coppice cycles and high yield to input ratio (Sims and Venturi, 2004). To alleviate the conflict between food and biofuel production (Rockwood et al., 2004; Edrisi and Abhilash, 2016), SRC plantations are typically grown on marginal lands, which are inadequate for high productivity crop growth (Herr and Carlson, 2013), thus generating an income without the need for a land remediation period (Paulson et al., 2003).

Short rotation coppice *Populus* clones are selected from world-wide breeding programs based on interspecific hybridization, whereby black poplar (*Populus nigra* L.) is a fundamental parental pool (Stanton et al., 2013); being widely and naturally spread in Europe, typically associated with riparian ecosystems, and characterized by large phenotypic and genetic variability (van der Schoot et al., 2000; Richardson et al., 2014; DeWoody et al., 2015). Moreover, *P. nigra* has been thoroughly studied due to its numerous adaptive characteristics, including easy clonal propagation, good coppicing ability, resistance to pathogens and parasites (Benetka et al., 2012), prolonged growing season (Rohde et al., 2011), and high plasticity in response to environmental conditions (Chamaillard et al., 2011). Breeding strategies based on recurrent selection and testing are frequently implemented for gradual population improvement (Neale and Kremer, 2011). Acceleration of the *Populus* domestication is also expected through recurrent intraspecific crossing and higher order species mixes, yet to be supplemented by genomic selection, association genetics, and genetic engineering (Harfouche et al., 2012). While first-generation hybridization (F_1_) is adopted to obtain heterosis for growth rate (Stettler et al., 1988), advanced generation breeding, such as F_2_ hybridization, among closely related *Populus* species, has proved to be an efficient strategy toward genetic improvement (Stanton et al., 2010).

Field-grown trees are routinely exposed to environmental stress and are likely to experience unprecedented rises in temperature and increases in the frequency and severity of summer drought episodes in the future (Lindner et al., 2014; IPCC, 2014). The physiological responses to drought are complex and traits vary in their importance depending on intensity, duration, and timing of the drought (Bréda and Badeau, 2008; Tardieu and Tuberosa, 2010; Harfouche et al., 2014). These traits present as reduced leaf size, decreased leaf growth rate, lowered stomatal aperture and density, reduced stomatal conductance (*g*_s_), and altered patterns of root development (Tardieu and Tuberosa, 2010). Furthermore, inside the leaf, prolonged drought periods reduce CO_2_ assimilation rates and the extra energy dissipation, with a consequent increase in reactive oxygen species production, leading to leaf senescence and yield loss (Pintó-Marijuan and Munné-Bosch, 2014). Physiological and molecular studies on drought tolerance in *Populus* have shed light on the considerable divergence in response to water deficit between different genotypes (Marron et al., 2002; Monclus et al., 2006; Street et al., 2006; Huang et al., 2009; Regier et al., 2009; Cocozza et al., 2010; Viger et al., 2016).

Plant tolerance to abiotic stresses is an ambiguous concept, even after distinguishing different strategies such as avoidance, tolerance, and escape (Levitt, 1972). Depending on their genetically dictated molecular and physiological attributes, plants budget their water in very different ways, along a continuum that ranges from the water-conserving or risk-aversion behavior displayed by isohydric plants to the risk-taking behavior displayed by anisohydric plants (Tardieu and Simonneau, 1998; Moshelion et al., 2014; Sade and Moshelion, 2014; Attia et al., 2015).

In reduced water availability conditions, the relationship between *g*_s_ and leaf temperature has been utilized as a valid indicator of trees’ response (Chaves et al., 2003; Bréda et al., 2006; Jiménez-Bello et al., 2011; Costa et al., 2013; Rebetzke et al., 2013; Struthers et al., 2015). Therefore, relating the leaf temperature of individuals to the average value of a population exposed to similar environmental conditions may be indicative of the trees’ state of stress.

A major obstacle to a more effective dissection of plant response to drought is the difficulty in properly phenotyping in a high-throughput fashion. To relieve a phenotyping bottleneck, phenotypic traits should be turned into quantifiable, objective, and consistent measures. Furthermore, automated and high-throughput phenotyping (HTP) on large-scale plant populations is expected to increase the probability of detecting crucial traits and, thus, identifying effective genotype-phenotype relationships (Goggin et al., 2015). HTP envisions a suite of strategies to speed up the phenotyping process and maximize the number of studied plants per experiment (Goggin et al., 2015). These methods enable automated, non-destructive, and non-invasive screening of high dimensionality populations, and thus, allowing the same plants and their responses to changing environmental factors to be monitored throughout their life cycle (Fahlgren et al., 2015a). To facilitate data interpretation, HTP platforms often involve observations in controlled conditions, such as growth chambers and greenhouses. However, plant performance in highly controlled conditions is poorly correlated with breeders’ target commercial and real-world environments (White et al., 2012; Araus and Cairns, 2014; Deery et al., 2014; Ghanem et al., 2015; Izawa, 2015; Poland, 2015). With regards to the specific case of drought, phenotyping under controlled conditions is highly challenging. In fact, the declining soil moisture content and increasing mechanical impedance typical of droughts are difficult to replicate in pots that are much smaller than the volume of soil available in the field (Cairns et al., 2011; Passioura, 2012). This fact may result in fast plant response through stomatal closure, which may, in turn, mask slower adaptive processes.

High-throughput phenotyping approaches seek to gather remote information; however, close-range (proximal) sensing is frequently required to provide the adequate resolution to decipher phenotypic traits (White et al., 2012). Proximal HTP combines robotic technology and imaging to enable high-dimensional phenotype screening and capture. Unmanned aerial vehicles (UAVs) equipped with cameras and sensors are proximal remote sensing that bridge the gap between time consuming ground-based measurements and satellite/airborne observations (Gago et al., 2015). Compared to traditional ground-based techniques, UAVs enable rapid and non-destructive measurements. They also offer much quicker turnaround times than satellites at competitive costs (Berni et al., 2009). In terms of spatial resolution, different from satellites, UAVs allow the acquisition of images whose pixels are significantly smaller than objects of interest, thus minimizing the bias effect due to background intensity (mixed-pixel problem) (Jones and Sirault, 2014). In contrast to aircrafts, such as manned helicopters, UAVs can safely hover at low altitudes in the proximity of plants, thus allowing high resolutions at low costs (White et al., 2012). In light of such advantages, UAVs are expected to open new avenues in field-based phenotyping of multiple stress traits and large populations rapidly, precisely, and accurately.

With regards to the evaluation of drought response, conventional ground-based methods typically require *g*_s_ measurements, which involve time consuming contact with leaves (Maes and Steppe, 2012; Costa et al., 2013). Alternatively, based on the relationship between *g*_s_ and leaf temperature, UAVs have been furnished with thermometers and thermal infrared (TIR) cameras to capture images of large-scale populations (Berni et al., 2009). TIR cameras show great potential in phenotyping as thermal images contain spatially distributed information about the energy emitted from body surfaces, such as plant leaves. Thermal images can be used to detect the state of stress due to drought and indirectly estimate *g*_s_ through the leaf energy balance equation (Jones, 1992, 1999). Since canopy temperature (*T*_c_) has long been recognized as a measure of plant water status (Jones et al., 2009), UAVs mounting sensors have been used for mapping drought response of agricultural crops at 20–40 cm spatial resolution (Sepulcre-Canto et al., 2006, 2007; Zarco-Tejada et al., 2012).

Although imaging has revolutionized plant phenotyping through the early and quantitative detection of plant traits in objective terms (Dhondt et al., 2013; Goggin et al., 2015), massive image data handling and processing remains the rate-limiting step in HTP (Fahlgren et al., 2015b). Image post-processing may include several steps, such as calibration and undistortion (Berni et al., 2009; Zarco-Tejada et al., 2013; Araus and Cairns, 2014), which should be automated in data management pipelines to boost HTP. Within data post-processing, the identification of objects of interest in images against other objects and the background (segmentation) (Jähne, 2005) is often the most critical step (Dhondt et al., 2013). In phenotyping studies, segmentation algorithms have been developed for plant three-dimensional measurements (Chéné et al., 2012) and to estimate crown architecture parameters (Díaz-Varela et al., 2015). For imaging to substantially contribute to HTP, standardized semi-automated image processing tools should be introduced and complemented with ground-truthing through well-established point measurement sensors (Fahlgren et al., 2015b).

Here, we developed a novel methodology for field HTP (HTFP) of drought stress in a *P. nigra* F_2_ partially inbred population using TIR images recorded from a UAV platform. The objectives of this study were to establish a field-scale HTP procedure to rapidly and precisely examine drought stress in trees; to provide objective image-based tools and statistical protocols to quantify phenotypic traits of moderately stressed and non-stressed trees; and to use HTFP to identify promising drought-tolerant genotypes for potential use within *Populus* breeding programs.

## Materials and Methods

**FIGURE 1:**
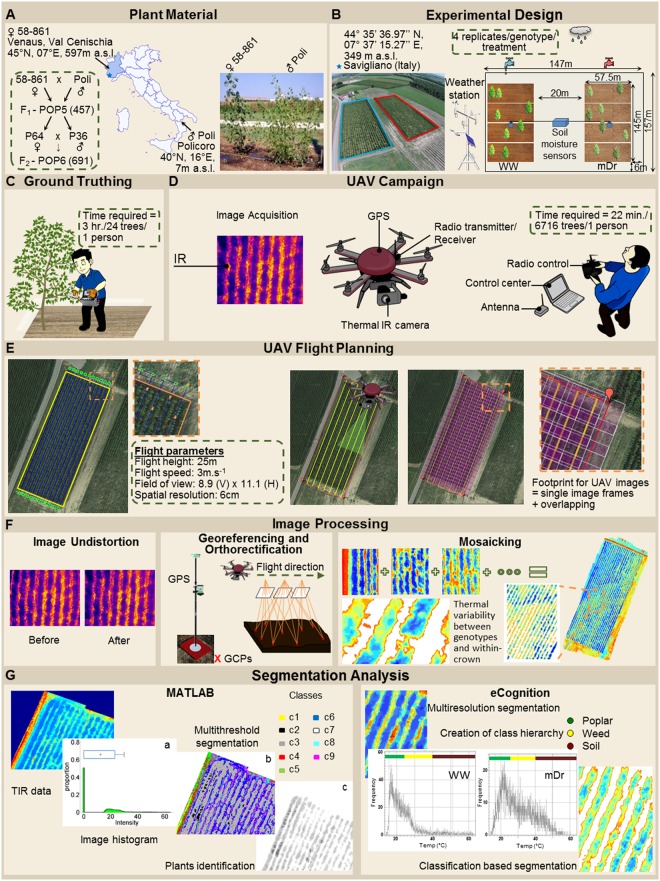
Workflow of the high-throughput field phenotyping (HTFP) methodology. **(A)** Plant material includes intraspecific *Populus nigra* full-sib F_2_ partially inbred population (POP6) obtained from the controlled cross between two *P. nigra* parents, P64 and P36. These parents were selected from an F_1_ breeding population (POP5) of 457 genotypes, obtained from an intraspecific *P. nigra* controlled cross between genotypes 58-861 and Poli. **(B)** Two adjacent plots were developed in (Savigliano (Italy) to host POP6 genotypes exposed to well-watered (WW) and moderate drought (mDr) stress conditions. In a plot, WW conditions were maintained, whereby water lost during the day through tree evapotranspiration (*ET*_c_, mm) was daily restored via drip irrigation. In the other plot, mDr conditions were maintained by withholding irrigation. In each plot, soil water content was daily monitored through a time domain reflectometry SM150 Soil Moisture Sensor installed at 50 cm underneath the soil surface. **(C)** Leaf stomatal conductance *g*_s_ was collected using a dynamic diffusion porometer. Measurements were taken on three biological replicates per treatment on each parental genotype (24 trees). **(D)** An unmanned FlyNovex^®^ multi-copter was integrated with a FLIR A35 TIR (thermal infrared) camera. The UAV campaign allowed for capturing TIR images of both experimental plots. **(E)** The flight mission was planned using the open source autopilot Mission Planner. The UAV was flown in the autonomous mode at a nominal speed of 3 m/s. **(F)** Fish-eye undistortion, image orthorectification, georeferencing, and mosaicking were performed using 16 ground control points (GCPs) captured with a global positioning system (GPS). **(G)** Canopy identification was achieved through two alternative automatic image segmentation approaches (an in-house algorithm in Matlab and eCognition).

The workflow of the developed methodology is illustrated in **Figure 1**. The HTFP method involves the following four steps: (1) UAV-based thermography to capture tree *T*_c_; (2) semi-automatic georeferencing, orthorectification, and mosaicking of TIR images; (3) tree canopy identification against bare soil through two independent image segmentation approaches; and (4) ground-truthing validation of UAV-based thermal data with *g*_s_ data.

### Field Experiments

#### Plant Material and Experimental Design

POP6 is a full-sib F_2_ partially inbred population consisting of 691 genotypes obtained from an intraspecific controlled cross between two *P. nigra* parents, P64 and P36. Parents have been randomly selected from an F_1_ breeding population (POP5) of 457 genotypes, obtained from an intraspecific *P. nigra* controlled cross between genotypes 58-861 and Poli (**Figure 1A**). Such genotypes originated from natural populations in divergent environmental conditions: 58-861 is from cold/wet climates typical of Val Cenischia (Northern Italy; 45°09′N, 07°01′E, 597 m above sea level), whereas Poli originates from warm/dry climates typical of Policoro (Southern Italy; 40°09′N, 16°41′E, 7 m above sea level). These genotypes are characterized by contrasting responses to water stress (Regier et al., 2009; Cocozza et al., 2010).

In January 2012, F_2_ genotypes were used to establish a stool bed and produce 1-year old material. Such material was harvested in January 2013 to produce hardwood cuttings. A large number of hardwood cuttings (20 cm long) was obtained per each genotype, and cuttings whose diameter was within 3–4 cm and with a large number of intact buds were labeled and stored at +4°C.

The experimental field-scale plots were located in Savigliano (Northern Italy; 44°35′36.97″N, 07°37′15.27″E, 349 m above sea level), and were established in April 2013. Two adjacent plots (1.67 ha of total extension) were developed to expose POP6 genotypes to two different water treatments (see Section “Water Treatment”) (**Figure 1B**). Each plot featured a completely randomized block design with four blocks. A single hardwood cutting per genotype was randomly assigned to each block to minimize variability attributable to eventually uncontrollable environmental factors (such as soil composition and fertility). Differently, for P64, P36, 58-861, and Poli, four cuttings were replicated per each block to ensure larger data availability. Cuttings were planted at a distance of 2.5 m × 1 m, between and within rows, respectively. In addition, the border effect, i.e., trees planted at external locations of plots display better growth conditions, was minimized by planting a double border row of *P. nigra* cv. Jean Pourtet around the sides of each plot. Therefore, a total of 6716 trees were planted in the experimental plots.

In March 2013, multiple sprouts were thinned to a single stem per stamp, choosing the most vigorous. During the first growing season in 2013, drought treatment was not applied to ensure a homogeneous root system development and, thus, minimize effects on shoot growth due to different cutting dimensions. In the same growing season, both plots were regularly irrigated with a drip irrigation system during dry periods, and weeds were controlled using mechanized and chemical practices. In February 2014, experimental plots were coppiced, and for the second growing season in 2014, plots were managed similarly to 2013. In February 2015, trees were again coppiced and, in March 2015, re-sprouts were thinned to a single stem. Finally, due to a mortality of 12.7% of the original trees planted in 2013, 4603 plants (503 genotypes) were available at the beginning of the drought experiment in July 2015.

#### Weather and Soil Condition Analysis

Weather data at the experimental setup were obtained from a meteorological station managed by Agency for Protection of Environment – Piemonte (ARPA-Piemonte^1^) and located at 10 km from the experimental plots. Observations gathered from 1994 to 2013 indicate an annual mean temperature of 11.8°C and a total annual rainfall of 740 mm at the site. According to Köppen-Geiger classification (Kottek et al., 2006), climate was classified as Cfb, that is, warm temperate, fully humid and with warm summers. During the experiment in 2015, a 2 km distant meteorological station (Delta-T Devices Ltd., United Kingdom) was also used to record hourly air temperature (*T*_air_), air humidity, and precipitation.

Soil samples were collected from the middle of plots to quantify soil texture and to estimate field capacity and permanent wilting point. These samples were taken from topsoil down to a maximum depth of 0.5 m. The soil type was a sandy loam (50.5% sand, 35.5% loam, and 14.0% clay), using the United States Department of Agriculture soil taxonomy. Soil field capacity and permanent wilting point were estimated to 34 and 9.5%, respectively. Soil texture was estimated using gravimetric analysis, whereas soil field capacity and permanent wilting point were evaluated using the pressure-based method (Richards and Weaver, 1944).

#### Water Treatment

Response to drought stress was investigated by exposing POP6 to different water regimes for a period of 26 days, from 2nd July 2015 (day of the year; DOY 183) to 28th July 2015 (DOY 209). In one plot, well-watered (WW) conditions were maintained, whereby water lost during the day through tree evapotranspiration (*ET*_c_, mm) was daily restored via drip irrigation, see **Figure 1B**. Water provisioned through drip irrigation was estimated based on site-specific reference evapotranspiration (*ET*_0_, mm) and on *Populus* crop coefficient (k_c_). *ET*_0_ was found according to FAO-56 Penman-Monteith equation (Allen et al., 1998), and k_c_ was set to 0.84 in July and to 1.21 in August (Guidi et al., 2008). In the other plot, moderate drought (mDr) conditions were established by exposing plants only to natural rainfall. Drought was imposed by withholding irrigation from DOY 183, and monitoring the progressive reduction of soil moisture until a pre-wilting (i.e., sub-lethal) level of soil water content was achieved.

In each plot, soil water content was daily monitored through a time domain reflectometry SM150 Soil Moisture Sensor (Delta-T Devices Ltd., United Kingdom) installed at 50 cm underneath the soil surface. Four measures per day were recorded using a DL6 Data Logger (Delta-T Devices Ltd., United Kingdom), and the daily average soil water content was estimated. Such system allowed controllability over the experiment, by ensuring that WW and mDr conditions were maintained in the plots. During the drought experiment, daily average *T*_air_ (°C), daily rainfall (mm), daily water deficit (mm), and soil water content expressed as percentage of water field capacity (%FC) in WW and mDr plots were reported (Supplementary Figures S1A–C). Soil permanent wilting point is reported as a percentage of the water field capacity (%FC = 27.9%). Daily water deficit was calculated as the difference between precipitation and *ET*_c_, according to Cantero-Martínez et al. (2007).

### Data Acquisition and Processing

#### UAV Campaign

An unmanned FlyNovex^®^ multi-copter (FlyTop, Italy) was integrated with a FLIR A35 TIR camera (FLIR Systems, United States), see **Figure 1D**. FlyNovex^®^ is a versatile and powerful (24V-6S motors) hexacopter (120 cm diagonal size) with a highly resistant carbon fiber frame and offering a 7 kg take-off weight. Its maximum transmission distance is 2 km and its maximum flight time is 20 min. The FLIR A35 was equipped with a 9 mm f1.25 lens. Its thermal sensitivity is less than 0.05°C at 30°C and the camera enables measurements in the range -25°C to +135°C. The image sensor is a Focal Plane Array (FPA) based on uncooled microbolometers with a spectral response in the range 7.5–13 μm. The camera field of view is equal to 48° (horizontally) × 39° (vertically), its resolution to 320 pixels × 256 pixels, and its spatial resolution to 2.78 mrad. The camera captures images at an acquisition frequency of 60 Hz.

Pictures are stored as 14 bit digital raw images. The camera is radiometrically calibrated, and its high accuracy and pixel-to-pixel sensitivity circumvent the need for ground infrared calibration targets and temperature correction during post-processing (Deery et al., 2016; Gómez-Candón et al., 2016). The camera is controlled by an embedded computer (Pico PC with a Cortex 9 processor) that stores raw images on an internal micro SDD memory card for the entire duration of the flight.

A total of 16 highly visible targets were positioned along the borders of the experimental plots (8 targets per plot were located at the corners and in the middle of each side) and used as ground control points (GCPs). The targets were used for georeferencing thermal images. A Real-Time Kinematic global positioning system (GPS; Leica Geosystems, Switzerland) GS08plus model with an accuracy of 3 mm was used for capturing GCP locations.

#### Flight Plan and Thermal Imaging

The flight mission was planned using the open source autopilot Mission Planner (APM Mission Planner, United States). The UAV was flown in the autonomous mode (GPS-waypoint navigation) at a nominal speed of 3 m/s. Experimental plots were scanned during two 11-min flights conducted on 28th July 2015 under stable cloudless and low-wind conditions. To ensure similar solar illumination angles and consistent proportions of sunlit and shaded leaves (Deery et al., 2014), flights were performed at 13:41 local time above WW and at 14:30 local time above mDr.

To capture the experimental plots in a single flight pass while maximizing image resolution, flight plan consisted of transects parallel to the plant rows, see **Figure 1E**. A ground station processed the UAV safety manual control and sent telemetry data (position, attitude, and status data) through a radio link at 2.4 GHz to a laptop computer. This communication link also allowed operation of the onboard TIR camera.

Thermal images were recorded at an elevation of 25 m from ground level, thus yielding an 8.9 (vertical) m × 11.1 (horizontal) m and a pixel size of 6 cm × 6 cm. Such a resolution is within POP6 average leaf area (46.47 cm^2^ or 1.3 pixels, unpublished data). Flying at such an elevation minimizes image distortions due to atmospheric effects. The selected flight plan enabled capture of thousands of high quality single images presenting 30% overlap and 50% sidelap. Since images captured during take-off, landing, and flight maneuvers were discarded from further processing, acquisition of images took a few minutes per experimental plot.

#### Stomatal Conductance Ground-truthing

To validate the UAV-based approach, we studied the relationship between ground-based midday stomatal conductance (*g*_s_, mmol m^-2^ s^-1^) and *T*_c_ collected from the UAV for selected genotypes, see **Figure 1C**. At the same time of UAV flights, we collected abaxial *g*_s_ data using a dynamic diffusion porometer (AP4, Delta-T devices Ltd., United Kingdom). Measurements were taken on three biological replicates per two water treatments on each of the four parental genotypes (3 × 2 × 4 = 24 trees). On each tree, two *g*_s_ technical replicates were taken on the first sunlit fully expanded leaf from the canopy top. For each plot, before measurements, the porometer was calibrated according to the experimental site *T*_air_ and humidity. Instrument calibration, *g*_s_ measurements, and moving within and between plots required approximately 3 h.

#### Thermal Image Processing

We collected a total of 7836 thermal images (“.fff” files) during the UAV campaign. Given the high overlap and sidelap, one frame every 20 (392 images) was converted to radiometric “.jpg” through IRT Analyzer (Grayess, United States), and fish-eye undistorted through Adobe Photoshop CC (Adobe Systems, United States) (lens adjustment tool) by setting the camera focal length to 9 mm, see **Figure 1F**. Image mosaicking was performed with the Image Composite Editor software (Microsoft Corporation, United States), and radiometric mosaics were then converted to grid data with Surfer (Golden Software, United States). Mosaics were orthorectified and georeferenced with ArcGIS 9.2 (ESRI, United States). Orthomosaics were georeferenced by manually matching the surveyed 16 GCPs (**Figure 1F**).

**FIGURE 2:**
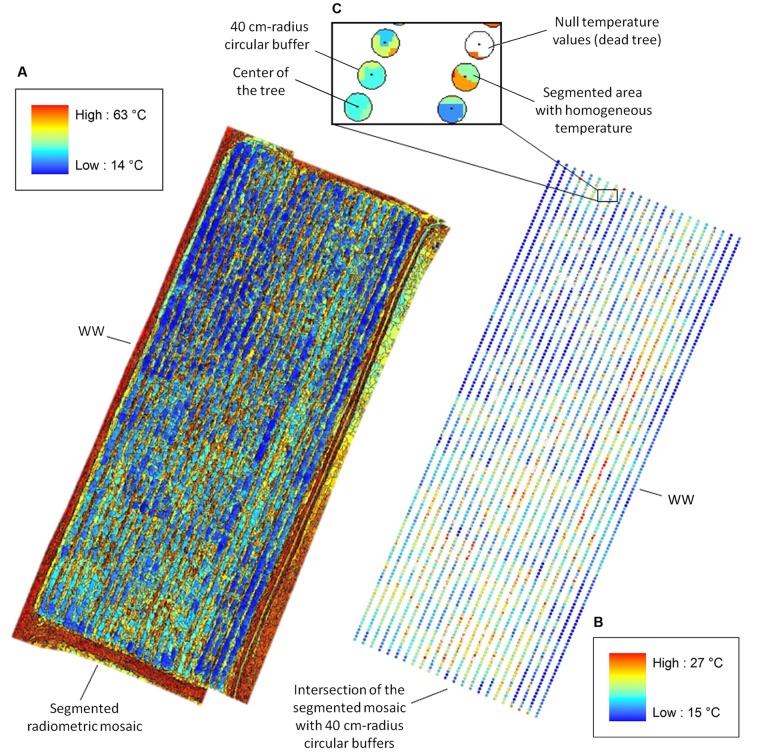
Canopy temperature (*T*_c_) extracted from segmented radiometric mosaics. **(A)** eCognition-based segmentation of the WW radiometric mosaic with temperature range between 14 and 63°C (temperature color bar on the top left). **(B)** Intersection of the segmented mosaic with 40 cm-radius circular buffers centered on the trees with *T*_c_ ranging between 15 and 27°C (temperature color bar on the bottom right). **(C)** View of the intersection for six WW trees. Pixels pertaining to “Soil” and “Weed” are assigned null temperature values (white areas) in the intersected buffer, whereas pixels relative to “Poplars” range from 15 to 27°C and display different colors for segmented areas at homogeneous temperatures. *T*_c_ for each plant is estimated by averaging values of pixels lying inside each intersected buffer.

Mosaics were processed to remove bare soil pixels (Jones and Sirault, 2014), and used to estimate average *T*_c_ (°C) for each tree. In particular, radiometric mosaics were combined with the position of the tree centers and spacing in the experimental plots (**Figure 2**). Firstly, radiometric mosaics were segmented to automatically identify regions depicting trees. Canopy identification was achieved through two independent semi-automatic image segmentation approaches (**Figure 1G**). We utilized the eCognition commercial software (eCognition Developer 9, Tremble Inc., United States) that is commonly adopted in image-based analysis for environmental applications. Further, we in-house developed a second segmentation algorithm in a Matlab environment (Matlab R2014a, The Mathworks Inc., United States).

In the eCognition segmentation, parameters such as canopy shape (set to 0.1), compactness (set to 0.5), and scale (set to 10) were used to identify trees. To address bare soil pixel removal, two pixel classes were introduced. One class included pixels at temperatures ranging from 15 to 27°C and from 14 to 28°C for WW and mDr, respectively (named “Poplar” in **Figure 1G**). Conversely, the second class consisted of pixels at temperatures lower and higher than the above ranges (named “Weed” and “Soil” in **Figure 1G**). We visually ascertained in the radiometric mosaics that the first class of temperatures was related to plants, and the second to soil and weed. Segmented areas “Weed” and “Soil” were discarded in the subsequent temperature extraction analysis.

In the alternative segmentation approach, Otsu’s threshold selection method was implemented on the entire radiometric mosaics (Otsu, 1979). Mosaic histograms were thresholded to obtain nine level-segmented images. The effectiveness of the segmentation was assessed through Otsu’s objective criterion (*N* = 0.99 in case of eight thresholds). Based on visual inspection of the mosaics, pixels at temperatures ranging from 15 to 27°C (WW) and from 14 to 28°C (mDr) were retained for estimating average *T*_c_.

After segmentation, pixels relative to soil and weed were assigned null temperature values in the radiometric mosaics. Then, 40 cm-radius circular buffers centered on the trees were intersected with the segmented mosaics (**Figure 2**). Since neighboring plants were spaced by 1 m within a row, such conservative buffer was selected to guarantee the precise identification of each plant canopy. Finally, *T*_c_ for each tree was calculated by averaging pixel values lying inside each intersected buffer.

#### Statistical Analysis

Phenotypic variance of a total of 503 genotypes was investigated using R software (R v.3.1.3, R Foundation for Statistical Computing, Austria). Due to natural replicates mortality since plot establishment, only genotypes with at least three replicates in both WW and mDr were retained in the analysis. Two-way ANOVA (ANalysis Of VAriance) inferential statistic procedure was used to describe the effects of genotype, treatment, and their interaction on total phenotypic variance observed in POP6. To respect ANOVA assumptions, the Box-Cox procedure (Box and Cox, 1964) was performed on the additive model to yield optimal data transformation, and Bartlett’s test was used to test the homogeneity of variance. When statistically significant differences among blocks were found, block effect adjustment was conducted to minimize the influence of competition among neighboring trees on genotype-specific response to drought conditions. Adjustment was performed according to Dillen et al. (2009), and it was repeated separately for each experimental plot.

The generalized linear mixed model was built to test differences among genotypes within each treatment and between the two treatments, and differences due to the genotype by treatment interaction (G × T). G × T aims at testing the consistency in the relative performance of genotypes grown in different conditions (White et al., 2007). Statistical significance was considered for *p*-values ≤ 0.05. To capture the proportion of total phenotypic variance due to genetic variation, broad-sense heritability (*H*^2^) was estimated for *T*_c_ in each treatment according to Sehgal et al. (2015):

$$H^{2}=\sigma_{G}^{2}/\left[\sigma_{G}^{2}+\left(\sigma_{\epsilon }^{2}/r\right)\right]$$

Here, $\sigma_{G}^{2}$ and σ_ε_^2^ are the genetic and residual variance components, respectively, and *r* is the average number of replicates for a genotype within treatment (i.e., 3.5).

Moreover, to provide a better estimation, *H*^2^ was calculated over combined treatments, taking into account the G × T effects on total variance:

$$H^{2}=\sigma_{G}^{2}/\left[\sigma_{G}^{2}+\left(\sigma_{G\times T}^{2}/n\right)+(\sigma_{\epsilon }^{2}/nr)\right]$$

Here, $\sigma_{G\times T}^{2}$ is the G × T variance component, *n* is the number of treatments, and *r* is the average number of replicates for a genotype in the experiment (i.e., 7.3).

Variance components were obtained using the restricted maximum likelihood (REML) procedure, with treatment as fixed effect, and genotype and G × T as random effects.

Finally, to evaluate the robustness of the independent segmentation procedures, differences between *T*_c_ datasets obtained with eCognition and Matlab, and the precision of both procedures was tested using the Student’s *t-*test and the Spearman’s rank correlation test. Statistical significance of the correlation coefficient (*ρ*) was considered for *p*-values ≤ 0.05.

#### Stress Susceptibility Index

*Populus nigra* genotype response to drought was dissected by computing the Stress Susceptibility Index (SSI) on UAV-based *T*_c_. Such an index has proved to be an efficient tool to classify plants according to their tolerance or sensitivity to water stress (Sánchez et al., 2015). SSI was calculated based on genotypic mean *T*_c_ according to Fischer and Maurer (1978):

$$SSI=[1-(T_{c}mDr/T_{c}WW)]/\left[1-\left(\overset{¯}{T_{c}}mDr/\overset{¯}{T_{c}}WW\right)\right]$$

Here, *T*_c_mDr and *T*_c_WW correspond to genotypic means under mDr and WW conditions, respectively, and 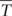
_c_mDr and 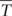
_c_WW correspond to POP6 means in mDr and WW conditions, respectively.

Negative SSI values correspond to a decrease in the genotypic mean *T*_c_mDr with respect to *T*_c_WW together with an increase in 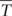
_c_mDr with respect to 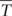
_c_WW. SSI values equal to 0 indicate consistent genotype means in mDr and WW conditions, regardless to POP6 mean responses. SSI values comprised between 0 and 1 indicate that *T*_c_ increase in mDr with respect to WW in the genotype is lower than *T*_c_ increase observed in the population. SSI values equal to 1 correspond to consistent deviations between mDr and WW conditions for both genotype and population means. SSI values greater than 1 indicate that *T*_c_ increase in mDr with respect to WW in the genotype is greater than *T*_c_ increase observed in the population. Therefore, genotypes whose index lies between 0 and 1 suggest an improved response to drought as compared to the overall population behavior.

## Results

### UAV-Based HTFP for Detecting Response to Drought

The UAV-based methodology allowed collection of high-throughput thermal data on experimental plots covering an area of 1.67 ha in only 22 min. While ground-truthing required 3 h to obtain *g*_s_ data on 24 trees, by performing only two low-elevation UAV flights, we were able to capture the response to drought of 6716 trees with a spatial resolution of 6 cm × 6 cm, that is, at POP6 sub-leaf definition. The methodology was simple to implement in the field: TIR camera required no infrared target-based calibration; standard GCPs were located in the experimental plots; and the UAV was autonomously navigated based on pre-planned mission (**Figure 1**). Data processing was almost fully automated: images were undistorted, mosaicked, and orthorectified with commercial user-friendly software. The supervision of an expert was mandatory to georeference the orthomosaics and to segment images. Image segmentation allowed for addressing the mixed-pixel problem and automatically identifying tree canopies through two independent algorithms. Both methodologies required an expert user to set parameters and classes based on visual inspection of the orthomosaics, and their implementation was computationally inexpensive. The extraction of tree *T*_c_ from radiometric orthomosaics for both treatments was executed within 1 h, and computational time devoted to statistical analyses was on the order of a few minutes.

**FIGURE 3:**
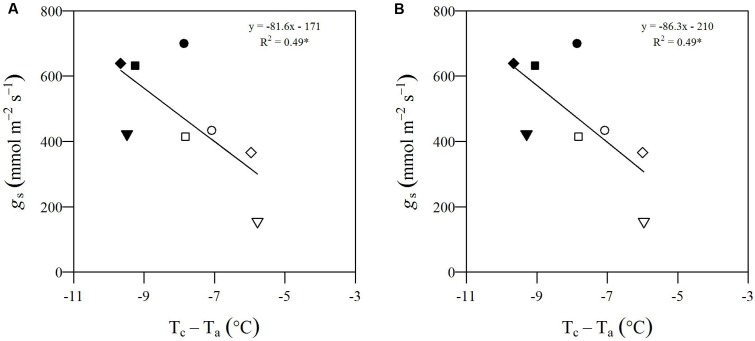
Relationship between *g_s_* (mmol m^-2^ s^-1^) and the difference between *T*_c_ and *T*_a_ (°C) for parental genotypes. **(A)** eCognition-based data and **(B)** Matlab-based data. For Poli (circle), 58-861 (triangle), P36 (square), and P64 (diamond), *T*_c_ and *g*_s_ are obtained by averaging measurements taken on biological replicates. Both sets of data are fitted with a linear function (*R*^2^ = 0.49). Data relative to mDr conditions are displayed as white symbols, whereas WW data are shown as black symbols. During the UAV flights, *T*_a_ was equal to 28.75°C. Statistically significant (*p*-value ≤ 0.05) regressions are indicated with the symbol ^∗^.

To validate UAV-based data, we utilized *T*_c_ of selected genotypes to recover the well-known inverse correlation between *g*_s_ and the difference between *T*_c_ and *T*_air_ (Farooq et al., 2009; Costa et al., 2013; Virlet et al., 2014). **Figure 3** illustrates the relationship between *g*_s_ and the difference between *T*_c_ and the air temperature at the time of the UAV flight (T_a_) for parental genotypes. They were chosen due to specific morphological and ecological traits, which were expected to lead to divergent drought responses. In **Figure 3A**, we show eCognition-based data, and, in **Figure 3B**, we report Matlab-based data. In both graphs, data relative to mDr conditions are found at high values on the *X*-axis and with *g*_s_ ranging from 154–434 mmol m^-2^ s^-1^ on the *Y*-axis, whereas lower WW data correspond to higher *g*_s_ values (ranging from 422 to 700 mmol m^-2^ s^-1^). Both data sets were fitted with a linear regression, statistically significant at an *R*^2^ of 0.49 (*p*-value < 0.05), suggesting that UAV-based thermal data accurately captured plant response to drought conditions. *T*_c_ and *g*_s_ mean values, with relative standard errors, are shown in **Table 1**.

**Table 1:** Phenotypic data for *T*_c_ and *g*_s_ for poplar parental genotypes.

	*g*_s_ (mmol m^-2^ s^-1^)		eCognition-based *T*_c_ -*T*_a_ (°C)		Matlab-based *T*_c_ -*T*_a_ (°C)	
			
Genotype	WW (Mean ± SE)	mDr (Mean ± SE)	WW (Mean ± SE)	mDr (Mean ± SE)	WW (Mean ± SE)	mDr (Mean ± SE)
Poli	700 ± 60	434 ± 16	-7.86 ± 1.33	-7.08 ± 0.73	-7.86 ± 1.33	-7.08 ± 0.70
58-861	422 ± 32	154 ± 6	-9.48 ± 1.25	-5.77 ± 0.36	-9.29 ± 1.44	-5.95 ± 0.18
P36	631 ± 95	414 ± 215	-9.25 ± 0.80	-7.82 ± 1.50	-9.05 ± 0.99	-7.82 ± 1.49
P64	639 ± 120	366 ± 45	-9.66 ± 0.69	-5.96 ± 1.21	-9.66 ± 0.69	-5.99 ± 1.17


*Mean values with standard errors (Mean ± SE) within each drought treatment for *g*_s_ (mmol m^-*2*^ s^-*1*^) and for the difference between *T*_c_ and *T*_a_ (°C).*

Remarkably, both segmentation approaches led to consistent relationships. To further assess the robustness of the independent segmentation methodologies, we computed the Student’s *t-*test on POP6 genotypic mean *T*_c_, separately for mDr and WW conditions. Notably, differences in data obtained with eCognition and Matlab were not statistically significant for both treatments (WW: *p*-value = 0.82 and mDr: *p*-value = 0.36). Moreover, on data sets obtained with eCognition and Matlab, we also evaluated the Spearman’s rank correlation test, and *ρ*-values of 0.98 (*p*-value < 0.001) were found for both WW and mDr conditions. Based on such strongly significant correlation, both segmentation methods showed consistent *T*_c_ estimations and, therefore, could be interchangeably utilized to extract *T*_c_ from thermal images. In the succeeding figures, we report results for eCognition data; genotypic mean *T*_c_ along with standard error and SSI values for both eCognition and Matlab are provided in the Supplementary Tables S1, S2.

### POP6 Response to Drought

We report results for 503 genotypes (out of the original 691, due to tree mortality) for eCognition and Matlab-based segmentations. We only retained genotypes with at least three survived replicates in both mDr and WW conditions.

**FIGURE 4:**
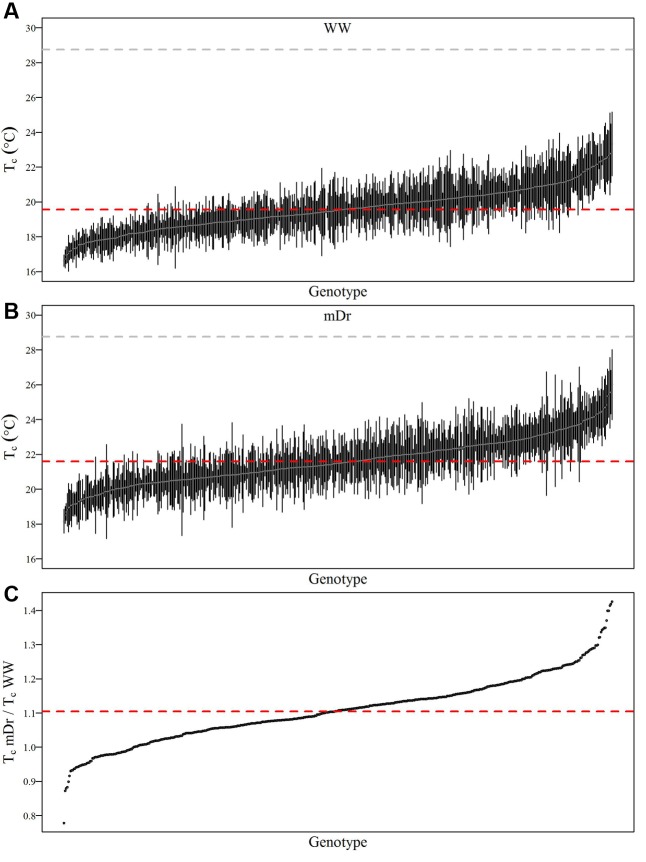
Average *T*_c_ (°C) for each genotype in WW and mDr. **(A)** Genotypic mean *T*_c_ (gray circles) and standard error (black bars) in WW conditions. **(B)** Genotypic mean *T*_c_ (gray circles) and standard error (black bars) in mDr conditions. **(C)** Ratio of *T*_c_mDr to *T*_c_WW (black circles). Genotypes in **(A,B)** are ordered based on increasing *T*_c_. Genotypes in **(C)** are ordered based on increasing ratios. The dashed gray line and dashed red lines in **(A,B)** show *T*_a_ during the unmanned aerial vehicle (UAV) flights and population averaged *T*_c_, respectively. The dashed red line in **(C)** displays population averaged *T*_c_mDr/*T*_c_WW. A total number of 503 genotypes is shown in **(A–C)**.

To show within-population and within-genotype variability in *T*_c_, **Figure 4** displays genotypic mean *T*_c_ in WW (A) and mDr (B) conditions as obtained after segmentation with eCognition. In addition, in **Figure 4C**, we compared the genotypic response to drought by reporting the relative increase in *T*_c_mDr with respect to *T*_c_WW (*T*_c_mDr/*T*_c_WW). Genotypes were ordered based on increasing *T*_c_ (**Figures 4A,B**) and *T*_c_mDr/*T*_c_WW (**Figure 4C**). Dashed red lines show POP6 mean *T*_c_ (**Figures 4A,B**) and *T*_c_mDr/*T*_c_WW (**Figure 4C**).

In **Figure 4A**, a percentage of 49.3% of genotypes lies above the average *T*_c_ of 19.55°C. For Matlab, 48.4% of genotypes were found above a consistent average *T*_c_ of 19.55°C. Further, the standard error of each genotypic mean ranges from 0.05 to 2.86 for eCognition and from 0.05 to 3.24 for Matlab. In **Figure 4B**, a percentage of 46.3% of genotypes lies above the average *T*_c_ of 21.60°C. For Matlab, 47.4% of genotypes were found above a similar average *T*_c_ of 21.55°C. Further, the standard error of each genotypic mean ranges from 0.08 to 3.56 for eCognition and from 0.16 to 3.17 for Matlab. Genotypic mean *T*_c_ in mDr was on average warmer than in WW conditions. In WW settings, in eCognition, average *T*_c_ ranged from 16.62 to 23.33°C (Matlab: *T*_c_ ranged from 16.62 to 23.06°C). With regards to mDr settings, in eCognition, average *T*_c_ spanned from 18.16 to 26.00°C (average *T*_c_ in mDr ranged from 18.14 to 24.87°C for Matlab).

In **Figure 4C**, the average ratio is equal to 1.11 for both eCognition and Matlab. The percentage of genotypes lying above such value (up to a maximum value of 1.41) is equal to 49.7 and 48.6% for eCognition and Matlab, respectively. A total of 37.38% (Matlab: 38.49%) of genotypes are found between 1 and 1.11, whereas only 12.92% (Matlab: 12.90%) of genotypes have ratios ranging from 0.79 to 1.

**FIGURE 5:**
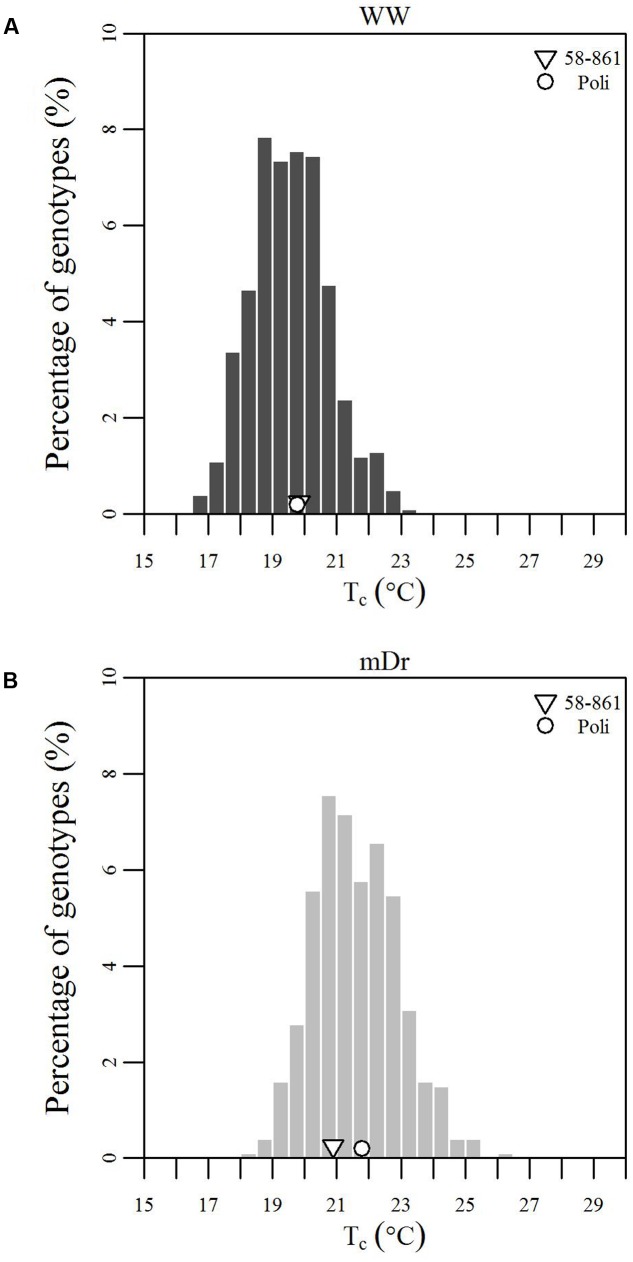
Frequency distributions of genotypic mean *T*_c_ (°C). **(A)** eCognition data for WW and **(B)** mDr conditions. Histogram bins are 0.5°C in width. The triangle and circle indicate genotypic mean *T*_c_ for 58-861 and Poli, respectively.

To inspect the frequency distribution of genotypic response to drought, in **Figure 5** we report histograms for genotypic mean *T*_c_ obtained with eCognition for WW (A) and mDr (B) conditions. Such frequency distribution is expected to provide insights into POP6 average response to treatments and also the population response with respect to 58-861 and Poli. Both graphs display an approximately symmetric distribution (skewness lower than 0.4) with a slightly platykurtic shape (kurtosis almost equal to 0). Similar data distributions were found for Matlab (**Table 2**). Further, we show average *T*_c_ for 58-861 and Poli genotypes. In WW, parental genotypes present a similar *T*_c_ (58-861 equal to 19.84 and 19.80°C; Poli equal to 19.76 and 19.75°C in eCognition and Matlab, respectively). Conversely, a greater difference in *T*_c_ between parental genotypes was found in mDr than in WW (58-861 equal to 20.88 and 20.93°C; Poli equal to 21.78 and 21.66°C in eCognition and Matlab, respectively). Different from parental genotypes, POP6 showed a large range of variation within WW and mDr treatments for both segmentation procedures. Indeed, for eCognition, average *T*_c_ ranged from 16.62 to 23.33°C in WW, and from 18.20 to 26.01°C in mDr. For Matlab, a very similar range of variation (with respect to eCognition) was found in WW (from 16.62 to 23.05°C), whereas a narrower range was observed in mDr (from 18.16 to 24.84°C).

**Table 2:** Frequency distribution shape parameters.

Segmentation Procedure	Treatment	Mean (°C)	Median (°C)	Kurtosis	Skewness
eCognition-based *T*_c_	WW	19.55	19.53	-0.13	0.27
	mDr	21.60	21.49	-0.01	0.35
Matlab-based *T*_c_	WW	19.55	19.48	-0.13	0.28
	mDr	21.55	21.48	-0.30	0.18


**T*_c_ Mean (°C), median (°C), kurtosis, and skewness values for histograms in **Figure 5**.*

To quantitatively assess the effect of the treatments on POP6, a two-way ANOVA was used to analyze *T*_c_ differences among genotypes, between treatments, and due to G × T interaction (**Table 3**). Notably, differences among genotypes were not statistically significant. On the other hand, differences between treatments were statistically significant. Finally, G × T interaction was not statistically significant for both eCognition- and Matlab-based *T*_c_.

**Table 3:** Two-way ANalysis Of VAriance (ANOVA) on eCognition and Matlab-based *T*_c_ (°C) in POP6.

Segmentation procedure	Source of variance	Degrees of freedom	Sums of squares	Mean square	*F*-ratio	*p*-value
eCognition-based *T*_c_^∗^	Genotype	498	0.01564	0.000031	1.056	0.211
	Treatment	1	0.02108	0.021077	708.437	<0.001
	G × T	498	0.01542	0.000031	1.041	0.276
	Error	2634	0.07836	0.000030		
	Total	3631	0.13050			
Matlab-based *T*_c_^∗∗^	Genotype	498	0.0751	0.00015	1.037	0.295
	Treatment	1	0.1038	0.10380	713.445	<0.001
	G × T	498	0.0765	0.00015	1.056	0.209
	Error	2642	0.3844	0.00015		
	Total	3639	0.6398			


*^∗^To respect ANOVA assumptions, individual eCognition-based *T*_c_ raw data were transformed by ˆ-1. ^∗∗^To respect ANOVA assumptions, individual Matlab-based T_c_ raw data were transformed by ˆ-0.5. Effects of genotype, treatment (well-watered, WW and moderate drought, mDr), and two-factor interaction (G × T) on *T*_c_.*

To address the relationship between genetic and environmental sources of variance, *H*^2^ of the *T*_c_ was estimated. Within treatment analysis resulted in very low *H*^2^ for both eCognition (WW: 0.09, mDr: <0.01) and Matlab-based *T*_c_ (WW: 0.10, mDr: <0.01). Similarly, very low *H*^2^ (0.015 in eCognition and <0.01 in Matlab) was observed for combined treatments.

### Selection of Putative Drought-Tolerant Genotypes

**FIGURE 6:**
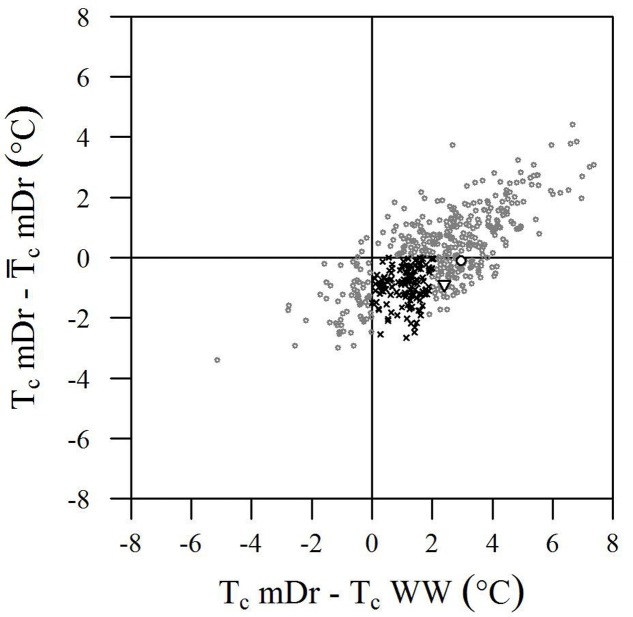
Biplot of genotype performance in response to drought stress. The difference between genotypic mean *T*_c_ (°C) and POP6 mean *T*_c_ in mDr is plotted against the difference between genotypic mean *T*_c_ in mDr and WW conditions. Genotypes are indicated with gray circles. White circles and white triangles correspond to Poli and 58-861, respectively. Black crosses indicate genotypes with (*T*_c_mDr – 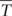
_c_mDr) less than 0 (genotypes are less stressed than the average POP6 response), and whose stress susceptibility index (SSI, defined in Section “Stress Susceptibility Index”) lies between 0 and 1 (the temperature increase in mDr with respect to WW in the genotype is lower than the temperature increase observed in the population). Such genotypes suggest an improved response to drought stress as compared to the overall population behavior.

Although the genotypic effect did not show a statistically significant influence on trait variance, we further analyzed *T*_c_ data to identify genotypes with improved drought tolerance. Genotype performance in response to drought is illustrated in **Figure 6**. Herein, the difference between single genotypic mean *T*_c_mDr and POP6 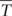
_c_mDr is plotted against the difference between genotypic mean *T*_c_mDr and *T*_c_WW conditions. Data show a positive correlation, where highly stressed individuals are located at the tails of the distribution and correspond to a less frequent behavior. Genotypes are mostly evenly distributed above and below the *X*-axis; however, stressed conditions lead to a majority of data points lying in the first and fourth quadrants.

From a drought-response perspective, the fourth quadrant in the biplot in **Figure 6** provides the most relevant information. Genotypes lying in the first and fourth quadrants [436 (86.68%) in eCognition and 438 (86.88%) in Matlab] present a *T*_c_mDr greater than *T*_c_WW. While genotypes in the first quadrant have a *T*_c_mDr greater than 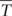
_c_mDr, those in the fourth quadrant show a *T*_c_mDr smaller than 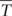
_c_mDr. A few genotypes display greater *T*_c_WW than *T*_c_mDr [data points in the second and third quadrants, 67 (13.32%) out of the total number of genotypes in eCognition and 66 (13.12%) in Matlab].

Due to mDr treatment, in genotypes found in the fourth quadrant (*T*_c_mDr > *T*_c_WW and *T*_c_mDr < 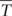
_c_mDr), *T*_c_ increased less than POP6 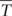
_c_mDr. This suggested the onset of acclimation mechanisms and, therefore, an improved response to drought stress with respect to the overall population. Among such drought-tolerant genotypes, 25% of the total number of tested ones (25.65% in eCognition and 25.79% in Matlab) presented an SSI ranging from 0 to 1, that is, the relative increase in *T*_c_ from WW to mDr conditions was lower than the increase observed on average in the population. Genotypes 58-861 and Poli were also located in the fourth quadrant; however, none of them had an SSI comprised between 0 and 1.

## Discussion

In this study, we developed a UAV-based HTFP approach to investigate the response of *P. nigra* to mDr conditions in the field. We assessed the effects of two water treatments on the observed phenotypic variance of an F_2_ partially inbred population of 503 genotypes. Notably, we remotely captured high resolution images, whereby image pixels were several orders of magnitude smaller than a single tree crown, and smaller than POP6 average leaf area. Such a resolution could be essential to uncover physiological differences within single crowns. A large leaf-to-leaf variability has been observed for *g*_s_ and leaf temperature in wheat (*Triticum aestivum*), which has led to low values of *H*^2^ when estimated on a single-leaf basis (Rebetzke et al., 2001, 2013). Gonzalez-Dugo et al. (2012) detected in mildly stressed almond (*Prunus dulcis*) that few areas within the crown had substantial stomatal closure while, in the rest of the crown, the stomata were still open and this increased heterogeneity of the *T*_c_. It has also been proposed that in cotton (*Gossypium* sp.) the variability of leaf temperature may provide important information about the degree of stomatal closure (Fuchs, 1990). In apple trees (*Malus pumila*) grown under drought conditions, the spatial variability of leaf temperature and *g*_s_ was higher for the whole crown than for the top crown (Ngao et al., 2017). By contrast, our UAV-based thermal imaging provides an ideal approach for the collection of the large number of individual leaf temperatures, which are necessary for methods based on temperature frequency distributions simultaneously in one image, rather than point-wise approaches for investigating tree response to drought. We also expect *g*_s_ to be consistently homogeneous in the upper canopy, that is, where *g*_s_ measurements were conducted herein.

High image resolution enabled the extraction of biologically meaningful data; in fact, plant leaves were attributed several image pixels, thus allowing accurate estimations of *T*_c_. Such a rapid and non-invasive UAV-based procedure is expected to highly benefit phenotypic-based assisted-mass selection in early generation screening in breeding programs for bioenergy purposes. UAVs could, indeed, be adopted to capture high resolution images over sparsely vegetated environments, such as orchards (Sepulcre-Canto et al., 2006; Sepulcre-Canto et al., 2007), or over very extended areas, such as natural forests (Torresan et al., 2016). While UAV-based phenotyping approaches have been already tested in agriculture, screening high dimensionality populations is a remarkable bottleneck in forestry applications and this is the first time that these measures have been applied to forest species.

Image processing at high speeds is a central challenge in the field of HTFP. Thermal imaging also allows leaves to be distinguished from the background. If done manually, however, the necessary image processing can be rather labor-intensive and may also be dependent on subjective image interpretation. Our images also identified thousands of tree canopies against background soil and weed through two independent semi-automated segmentation procedures. Segmentation was utilized to reduce the mixed-pixel problem by extracting contours of areas at consistent temperatures. Then, areas relative to trees were identified based on visual inspection of orthomosaics and retained for data processing. Alternative approaches, which are based on manually drawing tree canopies (Virlet et al., 2014), would be extremely time-consuming and may lead to user-biased results. In this study, we developed and standardized a semi-automated image-based analysis procedure and directly applied it on thermal orthomosaics. Without relying on RGB images, we evaluated the sensitivity of the method to image segmentation by experimenting with two independent algorithms. Remarkably, both segmentations led to statistically similar tree responses to drought, thus supporting the robustness of the methodology.

The level of stress induced by the treatment was fully captured through thermal images. This was indicated by the inverse linear relationship between *g*_s_ and *T*_c_. An increase in the difference of *T*_c_ with respect to *T*_a_ corresponded to a decrease in transpiration flux and, therefore, to a decrease in the ratio of actual to potential transpiration (Farooq et al., 2009; Virlet et al., 2014). Similar linear regressions with slightly higher *R*^2^ have also been observed for orange (*Citrus sinensis*) (*R*^2^ = 0.70–0.78) (Zarco-Tejada et al., 2012; Ballester et al., 2013a), persimmon (*Diospyros kaki*) (*R*^2^ = 0.46) (Ballester et al., 2013b), and almond (*Prunus dulcis*) (*R*^2^ = 0.59–0.66) (Gonzalez-Dugo et al., 2012), using UAV, small airplanes, and ground screening techniques. However, comparable results for forest tree species through UAV-based phenomics are still undocumented.

As already pointed out in a previous greenhouse study on early effects of drought on *P. nigra*, Poli tended to quickly respond to stress by closing stomata due to the fact that Poli is adapted to dry/hot climatic conditions. However, 58-861 reacted more slowly to drought as it is considered better adapted to cool and moist climates (Cocozza et al., 2010). This behavior could be explained by proved geographical and environmental gradients of *g_s_*, with higher *g_s_* values observed in northern proveniences of *Populus* sp. (McKown et al., 2013; Kaluthota et al., 2015). This motivates lower *T*_c_ values for 58-861 than Poli as observed in **Figure 5B**.

Due to drought stress, POP6 *T*_c_ increased and tended to the temperature of the environment as similarly seen in Mahan and Upchurch (1988). During the UAV flights, *T*_a_ was equal to 28.75°C; in WW conditions, the average difference between *T*_c_ and *T*_a_ was equal to -9.19°C. Similar differences (from 10 to 15°C) between air and leaf temperatures have already been demonstrated to be plausible (Jackson et al., 1981). As expected, such a difference decreased to -7.14°C in case of mDr conditions, when transpiration flux was reduced. These findings are consistent with previous drought studies (Zarco-Tejada et al., 2012; Ballester et al., 2013a,b). Also, in agreement with Ballester et al. (2013a,b), and Costa et al. (2013), the treatment resulted in a difference of 2°C between WW and mDr POP6 average *T*_c_. **Figures 5A,B** demonstrated that drought induced high phenotypic variability (large ranges of variation in frequency distributions). In fact, genotypic mean *T*_c_ followed a Gaussian distribution with a high degree of transgressive segregation in both treatments (thermal response of POP6 was extreme as compared to parental response). The high variability in F_2_ populations may be due to the transgressive segregation as was previously observed by Wu and Stettler (1994) and Rae et al. (2009) for growth-related traits in similar populations. This suggests that thermal response to drought is a quantitative trait controlled by several genes (polygenic trait) (White et al., 2007), and that *T*_c_ is under complex but repeatable genetic control (Rebetzke et al., 2013). In our study, the lack of statistically significant G × T interaction suggests that POP6 response is consistent between both treatments (i.e., stressed genotypes increase their *T*_c_, and genotypes with higher *T*_c_WW with respect to other genotypes also display higher *T*_c_mDr). Such consistency may facilitate the selection of genotypes of interest.

Poplar *T*_c_ response to water stress was further explored by investigating *H*^2^. A very low *H*^2^ was observed for POP6, due to a large residual error and a modest genetic influence on the phenotypic variance. Generally, *H*^2^ of a trait varies across different populations and environments (Griffiths et al., 2000), and it is overestimated when G × T interaction is significant (White et al., 2007). To the best of our knowledge, *H*^2^ estimation of *T*_c_ in forest tree species is still not reported. Yet, it was observed in the range from 0.05 to 0.91 in *T. aestivum*, with higher values of *H*^2^ obtained for multi-year and multi-environment trials (Rebetzke et al., 2013). Such a large variation supports the urge to conduct experiments in different environments and water limitation conditions. Moreover, it is noted that recurrent selection is optimal for improving traits with low *H*^2^ (Hopkins et al., 2009). Therefore, recurrent selection of the herein identified putative drought-tolerant genotypes would be a promising approach to accumulate favorable alleles in future crosses of POP6.

In addition, our technique aids in identifying genotypes that appear to be risk-takers versus those that are risk-averse; such an observation is known to occur in response to drought (Sade et al., 2012; Moshelion et al., 2014; Attia et al., 2015). In our experiment, Poli exhibits a risk-averse strategy by limiting transpiration and allowing *T*_c_ to increase, as opposed to 58-861, which appears to be a risk-taker genotype. In fact, Poli considerably increases its *T*_c_ from WW to mDr conditions. This response supports the fact that Poli could have a sensing mechanism that detects reduced water availability, and thereby closes stomata to avoid stress due to drought. Closed stomata at high light intensities could lead to photo-damage, and thus drought stress may become photo-oxidative stress, ultimately leading to biomass yield loss. In 58-861, a lower *T*_c_ increment (1.05°C in eCognition and 1.12°C in Matlab) than Poli was observed between treatments. This suggests that 58-861 could employ a less conservative strategy, and thus, being a putative risk-taker, which developed an anisohydric survival strategy to drought. This adaptive choice may be beneficial in moderate stress conditions; however, it may not provide any advantages in case of prolonged and more intense drought conditions. The likelihood of anisohydric genotypes to succumb earlier to drought would increase, and that of isohydric genotypes to more likely die of carbon starvation is a function of drought intensity and duration.

Our findings show that, among trees exposed to mDr conditions, 63 (13.32%) out of 503 genotypes were found in the third quadrant in **Figure 6**, thus indicating greater *T*_c_WW than *T*_c_mDr. This behavior may be attributed to lower-density canopies (with short and sparse branches and with a small canopy surface area covered with leaves) in WW than in mDr. Conversely, 129 genotypes (25%) were located in the fourth quadrant and displayed an SSI comprised between 0 and 1. Even though *g*_s_ measurements are required to validate this response, these POP6 genotypes could be considered more risk-takers, which supposedly maintain high *g*_s_ and lower *T*_c_. However, whether or not these risk-taker genotypes could be considered drought tolerant would depend on the period and severity of drought, and on the rate at which plants recover from drought exposure (Sade et al., 2012). For example, in Alvarez et al. (2007), anisohydric purple lovegrass (*Eragrostis spectabilis*) maintained higher *g*_s_ and CO_2_ assimilation, and showed better performance than isohydric miscanthus (*Miscanthus sinensis*) plants under optimal and mild-to-moderate drought conditions, but little difference was noted when both plants were subjected to severe drought. A greater research effort is currently needed to better understand the physiological mechanisms involved when plants tend to be risk-takers in stressed and risk-averse in non-stressed conditions.

Even if genotypic effect was not statistically significant, risk-taking genotypes accounting for 25% of the tested ones support that crossing genotypes with divergent morphological and ecological traits, such as 58-861 and Poli, resulted in augmented phenotypic variability (see also the large ranges of variation in **Figure 5**) in the early F_2_ POP6 generation. Higher-order crossings with differential breeding techniques based on selection are expected to lead to higher *H*^2^ and improved genetic gains. Promising genotypes were capable of controlling stomatal closure to raise their *T*_c_ by less than 2°C with respect to non-stressed conditions (SSI ranging from 0 to 1). Interestingly, in Rizhsky et al. (2002, 2004), upon similar drought conditions, leaf temperature increased by 2–5°C. This supports our finding that trees whose *T*_c_ increases by less than 2°C showed a better response within POP6.

The extensive HTP executed in this study was done in drought trials under natural field conditions. The results of this study prove that this methodology enabled high-throughput data analysis in the field upon fast and non-invasive acquisition of thermal images. Notably, this approach allowed efficient and precise phenotyping of large population of individuals at the same time, thus minimizing the influence of variable meteorological conditions on control (in this case WW) and treated (in this case mDr) trees. Image resolution was remarkably higher than in previous studies (Berni et al., 2009) and highly sufficient for accurately characterizing tree response even at crown and leaf levels. We indicate that flight elevation was also standardized to 25 m to ensure high image definition while guaranteeing that UAV rotor downwash did not disturb *T*_c_. Finally, the degree of automation employed in the developed HTFP method is highly desirable and will be widely adopted in field-based phenomics for forest trees genetics and genomics research. Since *T*_c_ is strongly related to *g*_s_, photosynthetic rate (Way and Oren, 2010), and leaf water potential (Testi et al., 2008), UAV-based thermal sensing may be an efficient, robust, and high-throughput tool for indirectly screening breeding populations, for selecting drought-tolerant, as well as risk-takers and risk-averse genotypes. This methodology is also promising toward monitoring the dynamics of stomatal movement in response to environmental stresses through rapid and repeated surveys. Improved knowledge of stress response will also be enabled through the synergistic integration of UAV remote sensing with more direct ground-based measurements (i.e., leaf fluorescence, leaf gas exchange, leaf water potential, leaf morphology, tree sap flow, and biomass production). Coupled with ground-penetrating radar, field-based phenomics will accelerate screening for drought tolerance by multi-scale analysis of root, crown, and leaf traits. Currently, UAVs have been outfitted with multispectral, hyperspectral, thermal, RGB, and near-infrared cameras, which are useful to evaluate plant response to stress (Costa et al., 2013; Shi et al., 2016). Future technological ameliorations will afford unprecedented measurements such as chlorophyll fluorescence imaging and 3D mapping using light detection and ranging (LIDAR) sensors, directly from drones, thus opening novel avenues in high-throughput stress phenotyping in forest trees. Promising examples of this vision can also include UAV integrated platforms equipped with multiple sensors for simultaneous data collection.

In breeding programs, this new aerial screening method may sensibly alleviate the effect of environmental factors such as varying exposure to sunlight according to time of the day, local climatic conditions, background radiation due to soil vegetative cover and soil water content, being precise, automated and quick. In fact, utilizing highly sensitive radiometrically calibrated TIR cameras may speed up tree screening, thus enabling repeated observations over longer periods of time and larger scale areas. Furthermore, augmenting breeders’ visual definition by selecting low flight altitudes may provide novel insights on the response to drought stress at the single leaf scale.

## Conclusion

In this study, we developed a high-resolution and high-throughput UAV-based phenomics method to investigate drought in the field. We applied this screening method to precisely and efficiently assess the response to drought of a *P. nigra* F_2_ population consisting of 503 genotypes planted on an area of 1.67 ha. We captured thermal images of stressed and non-stressed trees from an elevation of 25 m. We reconstructed thermal mosaics and extracted the average *T*_c_ by using two independent image segmentation techniques. We statistically analyzed genotypic temperatures, and identified putative drought-tolerant genotypes. This newly developed approach enabled high resolution thermal orthomosaics from quick UAV-based acquisitions and simultaneous screening of a significant number of individuals.

Two segmentation techniques for accurately analyzing TIR images, one developed in-house using Matlab, and another relying on commercial software eCognition, were successfully implemented to eliminate the mixed-pixel problem. They both led to consistent results, indicating that it is possible to use HTFP-based thermography for the screening of tolerance to drought stress in forest trees. However, considering the complexity of drought tolerance, we suggest it can only act as an accessory means in an active breeding program for drought by contributing significantly to phenotyping of tree response to water stress. Future studies will aim at extending the methodology to rapidly generate environmentally nuanced temporal measurements of physiological differences to contribute to predictive environmental stress response models. Through the approach developed here, candidate genes for drought stress responses can also be identified when this HTFP is combined with advanced genomics approaches.

Based on our analysis, a good correlation was found between UAV-based parental genotypic mean *T*_c_ and ground-truth *g*_s_ measurements. Genotypic mean temperatures exhibited a Gaussian distribution centered about parental behavior. Furthermore, the statistically significant differences observed between treatments were attributed to environmental conditions. Finally, based on SSI values, 25% of the population exhibited increase in temperature under mDr conditions by less than 2°C, and, thus, can be regarded as candidate drought-tolerant genotypes.

The use of UAV for field-based tree phenotyping under drought conditions is novel, but is expected to become an important tool for improving efficiency in forest-tree breeding for climate change. To date, no studies have been carried out attempting to use UAV-based HTFP for forest tree phenotyping in managed stress trials in which specific and well-defined conditions are imposed, and effectively deploy such platform in a breeding program. Thanks to its high resolution aerial imagery, accurate data processing, and relatively simple implementation, this HTFP shows promise as a precise and efficient tool for use in phenomics studies.

## Author Contributions

RL performed the ground-truthing, analyzed experimental data, and executed phenotypic and statistical data analyses. FT executed Matlab segmentation and, together with RL, developed results and wrote the manuscript. RS planned the airborne campaign, reconstructed thermal mosaics, and executed eCognition segmentation. SK contributed to develop the workflow and write the manuscript. GSM contributed to the original concept of the project. AH conceived the project and its components, designed and supervised the study, and revised the manuscript. All authors discussed the results, read and approved the manuscript.

## Conflict of Interest Statement

The authors declare that the research was conducted in the absence of any commercial or financial relationships that could be construed as a potential conflict of interest.

## Footnotes

1 http://www.arpa.piemonte.it
