# Supplementary material for: UAV-Based Thermal Imaging for High-Throughput Field Phenotyping of Black Poplar Response to Drought
Source: Front Plant Sci. 2017 Sep 27;8:1681. doi: 10.3389/fpls.2017.01681 (PMC5623950; doi:10.3389/fpls.2017.01681)
Supplement: Supplementary file 1 [file Presentation_1.PDF]

Supplementary Material

UAV-based Thermal Imaging for High-Throughput Field Phenotyping of Black Poplar Response to Drought

Riccardo Ludovisi, Flavia Tauro, Riccardo Salvati, Sacha Khoury, Giuseppe Scarascia Mugnozza, Antoine Harfouche*

*** Correspondence:** Antoine Harfouche: aharfouche@unitus.it

1 Supplementary Figure and Tables

1.1 Supplementary Figure

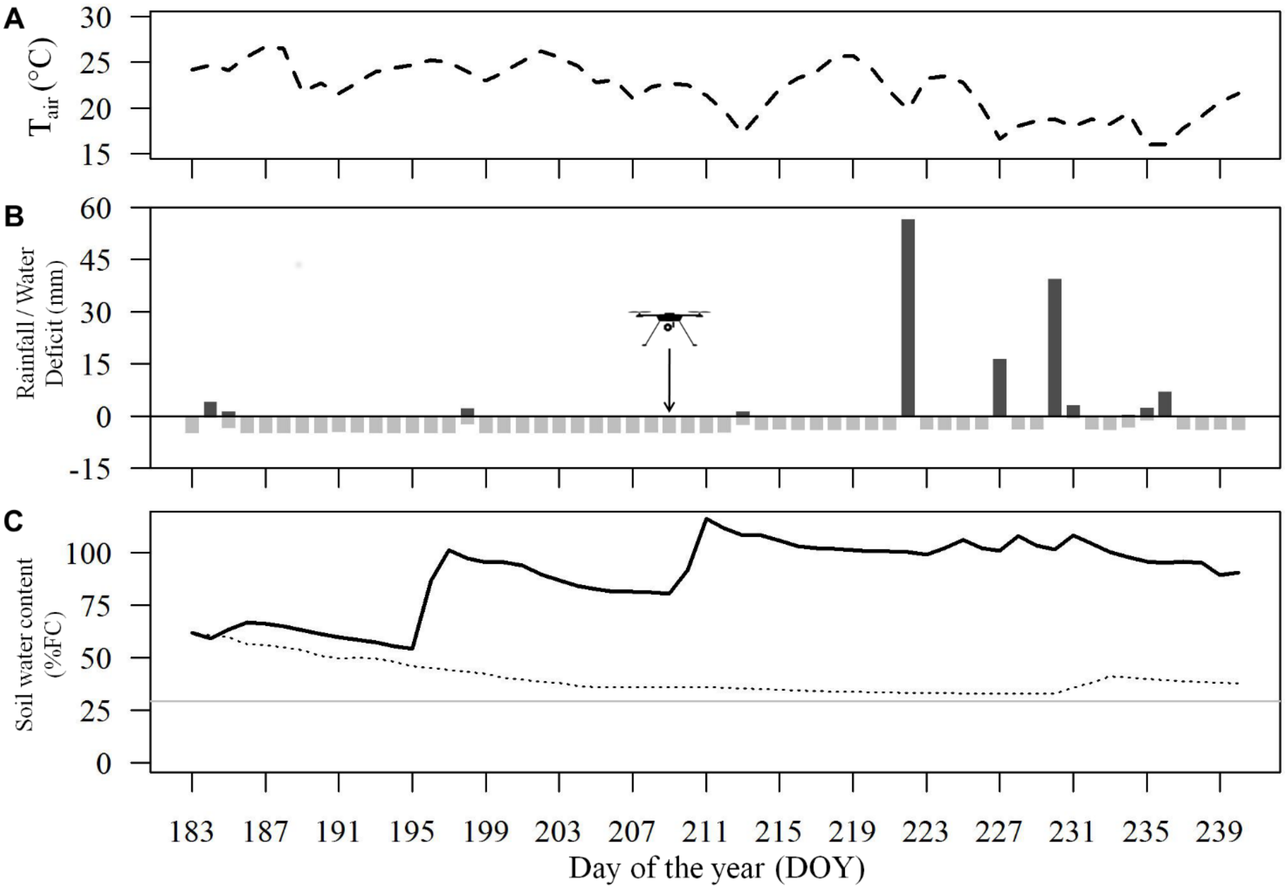

Supplementary Figure S1| Meteorological data and soil water condition. Meteorological data and soil water conditions observed from day of the year (DOY) 183 (2nd July 2015, beginning of water

withhold) to DOY 240 (28th August 2015). (A) Daily average air temperature (T_{air}, °C) (black dashed line), (B) daily rainfall (mm) (dark grey bar) and daily *P. nigra* water deficit (mm) (grey bar) observed during the water treatment experiment are shown. The drone icon and the arrow indicate DOY 209 (28th July 2015) when thermal data have been acquired. (C) Soil water content in WW (black solid line) and in mDr (black dashed line) is reported as percentage of water field capacity (%FC). The wilting point, equivalent to 27.9% of field capacity, is shown (grey horizontal line).

1.2 Supplementary Tables

Supplementary Table S1| Genotypic mean canopy temperature (T_c, °C) and standard error (SE, °C) for WW and mDr treatments. Data are based on eCognition segmentation. Values are computed based on the reported number of replicates (rep.). Stress Susceptibility Index (SSI) is also presented for each genotype.

	**WW**				**mDr**				
**genotype**	**T_c**		***SE***	**rep.**	**T_c**		***SE***	**rep.**	**SSI**
1	21.40	±	0.76	4	22.00	±	1.96	4	1.26
2	18.50	±	0.89	4	22.55	±	0.98	3	1.67
3	21.43	±	1.03	3	20.34	±	0.98	3	0.68
4	21.51	±	1.39	4	22.22	±	1.26	3	1.68
6	20.03	±	1.42	3	22.36	±	0.92	4	1.21
7	17.78	±	0.69	3	22.00	±	1.67	4	3.24
8	20.22	±	0.40	4	21.32	±	0.77	4	1.17
10	19.80	±	0.69	4	22.33	±	1.24	4	1.91
13	19.20	±	1.07	4	21.30	±	1.23	3	0.78
15	20.13	±	1.63	4	22.87	±	0.71	4	0.99
16	20.18	±	1.56	4	22.68	±	0.87	4	0.98
17	18.64	±	0.91	4	19.98	±	1.59	4	0.07
19	19.46	±	1.55	3	20.82	±	1.28	4	0.63
20	18.62	±	0.62	4	22.80	±	1.73	4	1.82
22	22.38	±	0.25	4	21.35	±	0.68	4	-0.97
24	21.48	±	2.24	3	22.76	±	2.04	3	1.12
25	17.81	±	0.78	4	20.30	±	0.35	4	0.96
26	21.12	±	1.75	3	22.34	±	2.39	3	0.32
28	20.19	±	1.41	3	22.38	±	1.83	4	-0.27
29	19.62	±	0.86	4	20.71	±	0.32	4	0.74

30	22.16	±	1.20	4	21.81	±	0.61	4	0.87
32	20.43	±	2.28	4	21.49	±	0.51	4	-0.28
33	20.92	±	1.35	3	20.92	±	1.32	4	-0.23
34	19.25	±	0.49	4	21.22	±	1.70	4	1.31
35	18.56	±	0.99	3	23.42	±	1.97	4	0.76
36	17.53	±	0.70	3	20.69	±	1.37	3	1.66
37	22.31	±	1.56	3	23.24	±	1.07	4	0.66
38	19.71	±	1.00	3	21.80	±	2.09	3	0.31
39	19.73	±	1.03	4	20.90	±	0.53	3	1.34
40	20.71	±	0.25	3	20.85	±	1.54	4	0.30
42	20.50	±	0.64	3	23.11	±	1.55	3	0.84
43	20.40	±	1.09	3	22.92	±	0.77	4	1.62
45	17.54	±	0.40	3	22.61	±	1.05	4	2.38
46	18.19	±	0.99	4	21.47	±	1.95	4	2.67
48	19.06	±	1.31	3	19.65	±	2.20	3	0.11
49	19.91	±	1.18	4	20.79	±	1.23	4	-0.13
50	18.53	±	1.21	3	22.67	±	0.96	4	0.75
51	18.27	±	0.55	4	22.35	±	1.41	4	2.19
52	20.51	±	1.02	3	23.39	±	0.69	4	1.73
53	20.32	±	1.00	4	23.10	±	1.13	4	0.97
54	19.71	±	1.31	4	20.85	±	1.07	4	0.53
55	19.05	±	1.54	3	20.35	±	0.56	3	0.42
56	21.35	±	0.83	4	20.82	±	3.02	3	-0.02
57	17.68	±	0.26	4	21.95	±	1.69	3	2.20
58	19.94	±	0.60	4	21.06	±	0.92	3	0.10
59	19.64	±	1.66	4	20.98	±	0.76	4	1.43
61	20.25	±	1.31	4	20.89	±	1.42	4	-0.23
63	20.19	±	1.40	4	20.49	±	1.66	4	0.44
65	21.31	±	0.74	4	20.68	±	0.57	4	0.60
66	19.60	±	0.28	4	21.22	±	0.83	4	1.00
67	20.65	±	0.66	4	22.10	±	1.81	4	1.30
68	19.15	±	0.98	4	22.25	±	0.99	4	1.50
71	18.64	±	1.34	3	20.65	±	0.65	4	0.75

73	19.63	±	0.59	4	22.24	±	1.49	4	1.16
75	20.05	±	0.94	4	22.28	±	1.14	3	1.23
76	19.99	±	1.14	4	21.11	±	0.82	4	1.82
77	17.86	±	0.93	3	20.97	±	1.47	4	1.31
78	18.83	±	1.00	3	23.04	±	1.09	4	1.60
79	21.74	±	1.34	3	22.72	±	1.03	4	0.20
80	19.41	±	1.37	4	20.83	±	1.65	4	2.31
81	19.32	±	1.61	3	22.33	±	0.54	3	2.59
82	17.39	±	0.41	4	19.89	±	0.94	4	1.35
83	19.09	±	1.60	3	21.22	±	0.75	3	0.78
84	19.39	±	1.01	4	21.93	±	1.96	4	0.60
85	21.38	±	0.71	4	20.80	±	1.50	4	0.85
87	17.84	±	0.92	3	20.08	±	1.28	3	0.91
88	21.03	±	1.50	3	22.47	±	1.43	4	0.54
91	18.88	±	0.65	4	23.61	±	1.96	4	2.01
92	20.53	±	1.37	4	19.38	±	0.09	3	0.76
93	20.12	±	2.42	4	20.49	±	1.39	4	-0.74
94	18.25	±	0.92	3	21.29	±	0.85	3	1.19
97	19.99	±	1.65	3	19.18	±	1.21	3	-0.13
98	21.22	±	2.10	4	20.79	±	0.73	4	1.22
100	20.77	±	1.24	4	20.26	±	1.77	4	0.42
101	19.55	±	0.90	4	20.83	±	0.82	4	0.30
104	20.18	±	1.73	4	21.83	±	1.55	4	1.57
106	19.17	±	0.50	4	21.95	±	1.75	4	2.32
107	19.20	±	0.99	4	22.76	±	0.74	4	1.53
108	17.91	±	0.59	3	19.43	±	0.28	4	0.65
110	18.07	±	0.64	3	20.73	±	0.39	3	1.51
111	18.45	±	0.48	4	20.72	±	1.01	4	0.45
112	19.18	±	0.67	3	20.35	±	0.83	4	1.36
114	18.97	±	1.35	3	21.98	±	0.48	3	2.12
116	18.77	±	1.30	4	20.55	±	0.97	4	1.14
117	20.80	±	1.78	3	19.78	±	0.94	3	-0.49
118	17.90	±	0.77	4	23.66	±	0.83	3	1.91

119	20.41	±	1.95	3	19.39	±	1.17	3	-0.51
120	20.59	±	0.91	4	19.54	±	0.77	4	0.79
122	19.95	±	1.89	3	21.51	±	1.59	4	1.65
124	18.50	±	1.34	4	21.33	±	1.53	4	1.29
126	20.64	±	0.93	4	21.93	±	1.17	4	0.60
127	18.58	±	0.71	4	20.58	±	1.13	3	-0.21
128	20.30	±	1.63	4	20.82	±	1.25	4	0.13
129	20.15	±	1.45	4	21.83	±	0.81	4	1.20
130	21.71	±	2.09	3	23.58	±	2.54	4	0.44
131	19.54	±	0.93	5	22.26	±	1.68	4	0.48
133	18.84	±	1.48	4	23.16	±	1.69	4	2.28
135	18.51	±	1.40	3	20.71	±	0.73	4	1.30
136	19.96	±	0.95	3	18.82	±	0.94	3	-0.45
138	19.30	±	1.47	4	22.05	±	0.54	3	1.83
139	20.11	±	1.64	3	22.49	±	1.75	4	1.25
141	20.73	±	1.88	3	21.17	±	0.90	4	1.53
142	19.31	±	0.99	4	21.94	±	0.66	4	1.45
144	18.96	±	1.33	3	21.92	±	1.81	3	0.27
145	17.84	±	0.61	3	21.17	±	1.67	4	0.68
146	17.87	±	1.17	4	19.29	±	1.18	4	1.35
148	20.17	±	2.48	3	21.79	±	0.93	4	1.99
150	19.11	±	1.51	3	20.52	±	1.67	4	1.49
151	20.71	±	2.26	4	23.31	±	0.75	3	0.49
152	19.63	±	0.62	4	20.46	±	0.61	4	0.91
153	21.21	±	1.97	3	20.33	±	1.02	3	-0.13
154	18.32	±	1.61	3	21.97	±	1.38	4	1.42
155	17.16	±	0.49	3	21.28	±	1.52	4	1.19
156	22.03	±	1.96	3	21.40	±	1.45	4	-0.39
158	20.55	±	1.10	3	20.28	±	0.93	4	-0.51
159	18.66	±	0.43	3	22.49	±	2.12	3	2.31
160	19.01	±	0.58	4	22.26	±	0.69	3	2.19
161	20.05	±	1.48	4	21.30	±	1.82	3	0.73
163	23.33	±	1.83	4	18.16	±	0.69	3	-2.10

164	18.36	±	0.94	4	22.61	±	2.09	4	1.45
165	21.15	±	1.20	3	22.72	±	1.53	4	0.24
166	20.28	±	2.46	3	20.33	±	0.45	4	0.82
167	19.00	±	1.10	4	24.25	±	1.12	4	2.86
168	19.50	±	0.88	3	21.15	±	0.85	4	1.31
169	17.20	±	0.45	3	20.57	±	2.04	3	0.50
170	18.60	±	1.25	4	20.63	±	0.80	4	1.41
171	22.58	±	1.56	3	23.45	±	0.95	4	0.97
172	19.64	±	1.41	4	19.86	±	1.27	4	1.46
173	18.79	±	1.51	4	20.31	±	0.87	3	0.52
175	19.85	±	1.72	3	20.64	±	0.69	4	1.20
176	22.04	±	1.89	4	20.67	±	0.76	4	-0.52
177	19.18	±	1.92	3	22.00	±	0.96	3	1.62
179	20.65	±	0.62	3	20.11	±	1.43	3	0.67
181	19.06	±	0.97	4	20.42	±	1.50	3	0.59
182	19.88	±	0.59	4	21.44	±	1.05	4	0.69
183	17.56	±	0.46	3	20.96	±	1.51	4	1.94
184	18.67	±	1.21	3	25.58	±	1.26	3	4.06
187	19.56	±	1.31	4	23.67	±	1.01	4	1.36
188	19.47	±	1.81	3	22.24	±	0.68	4	1.74
189	18.11	±	0.59	4	20.65	±	1.88	4	2.40
190	18.56	±	1.44	3	21.89	±	0.74	4	2.64
191	18.72	±	0.65	4	20.89	±	2.23	4	1.40
195	20.33	±	1.61	3	23.20	±	2.14	4	1.64
196	17.77	±	0.41	4	21.60	±	0.96	4	2.22
198	20.77	±	1.81	4	21.76	±	1.25	4	1.41
199	22.05	±	1.41	3	22.64	±	2.09	4	0.90
200	22.79	±	1.69	4	20.04	±	0.48	3	-1.15
201	20.32	±	1.07	4	23.09	±	0.67	4	0.80
202	19.97	±	1.65	4	20.13	±	1.63	3	1.33
203	18.86	±	0.94	4	21.83	±	1.03	4	2.14
206	19.84	±	1.05	3	22.46	±	1.67	4	0.66
207	19.91	±	1.08	4	19.57	±	0.91	4	0.50

208	19.91	±	1.14	3	23.17	±	1.10	3	0.32
209	18.77	±	0.64	4	20.55	±	1.11	3	0.69
210	17.31	±	0.61	4	24.48	±	1.99	3	2.94
215	19.74	±	1.88	4	20.81	±	1.37	3	0.85
216	19.90	±	1.78	3	22.09	±	1.42	3	0.49
218	20.86	±	1.09	4	21.02	±	2.02	4	0.56
219	22.51	±	1.37	3	19.87	±	1.50	3	-0.54
220	18.23	±	0.97	4	20.52	±	1.34	4	-0.15
221	21.04	±	2.00	4	22.64	±	0.53	4	0.95
222	19.32	±	0.96	4	18.69	±	0.48	4	-0.09
223	20.47	±	0.99	3	22.85	±	0.79	4	0.97
224	17.57	±	0.92	3	20.68	±	0.47	4	2.64
225	20.16	±	1.82	3	20.72	±	1.36	3	0.80
226	20.82	±	1.16	3	21.60	±	1.89	4	1.08
231	18.73	±	1.58	4	24.03	±	1.51	4	2.04
232	18.92	±	0.54	4	20.03	±	0.53	3	0.84
233	19.48	±	1.94	3	20.37	±	0.77	4	0.53
234	18.98	±	0.91	3	23.25	±	0.60	4	2.61
235	18.54	±	2.35	3	22.01	±	0.28	4	2.00
237	21.87	±	1.54	4	22.08	±	1.79	4	1.27
238	20.98	±	2.59	3	22.00	±	1.64	3	0.19
239	20.34	±	1.80	4	24.42	±	0.92	3	2.14
240	20.09	±	1.29	4	22.07	±	2.91	3	0.16
241	19.18	±	0.96	4	22.08	±	0.43	4	1.05
242	19.93	±	1.85	3	21.27	±	0.46	3	0.47
243	18.86	±	1.23	4	21.69	±	1.05	4	0.95
244	17.83	±	0.62	3	20.45	±	0.78	4	3.14
245	18.59	±	0.66	4	22.02	±	1.08	4	2.14
247	20.37	±	0.59	3	19.06	±	0.87	4	-0.54
251	20.72	±	1.32	4	21.57	±	1.20	3	0.14
252	20.88	±	1.53	4	19.65	±	0.60	3	-0.01
253	20.45	±	0.75	4	20.04	±	1.15	4	0.64
254	18.11	±	0.98	3	20.20	±	0.82	3	1.96

255	19.22	±	1.19	4	21.56	±	0.66	4	1.27
256	22.46	±	1.88	4	22.97	±	0.86	4	0.93
257	22.11	±	1.16	4	21.63	±	1.24	4	0.81
258	19.15	±	0.68	4	22.65	±	0.39	4	1.95
259	19.66	±	0.44	4	22.28	±	1.36	3	0.50
260	19.94	±	0.43	3	21.08	±	1.67	4	0.50
261	20.38	±	1.18	4	20.62	±	1.85	4	0.66
264	20.81	±	0.83	4	21.71	±	0.57	4	0.22
265	22.49	±	2.04	4	22.12	±	1.06	4	0.68
266	17.96	±	0.52	4	19.44	±	0.48	4	0.79
267	20.59	±	1.20	4	21.09	±	0.25	3	0.06
268	20.99	±	1.56	4	20.51	±	0.56	3	-0.45
271	19.87	±	1.49	4	24.12	±	1.19	4	2.23
273	19.57	±	0.91	3	22.72	±	1.53	4	-0.07
275	17.82	±	1.26	4	19.91	±	0.75	3	1.49
276	20.10	±	1.19	3	21.72	±	1.85	4	-0.44
277	19.27	±	1.87	4	23.72	±	3.30	3	1.95
278	21.95	±	1.54	3	22.44	±	1.16	4	-0.43
280	20.65	±	1.45	4	20.20	±	0.75	4	0.84
281	20.09	±	1.19	4	20.56	±	0.95	4	0.84
282	18.59	±	1.48	3	20.56	±	0.37	3	1.33
283	19.73	±	0.69	4	19.30	±	1.60	3	-0.19
287	17.54	±	0.74	3	22.55	±	1.86	4	1.44
288	18.51	±	0.56	3	21.40	±	1.98	4	0.72
289	19.86	±	1.22	3	19.64	±	0.90	4	-0.67
290	19.03	±	1.58	3	20.39	±	1.65	4	0.74
292	20.16	±	1.93	4	23.35	±	1.12	4	0.96
293	17.75	±	0.55	4	21.59	±	0.23	3	1.83
295	19.83	±	1.54	4	20.65	±	1.64	3	-0.60
296	18.20	±	0.64	3	19.58	±	0.61	4	0.70
297	20.33	±	0.70	4	21.31	±	1.02	4	0.32
298	18.84	±	1.46	3	19.95	±	0.22	4	1.76
299	20.55	±	0.81	3	24.09	±	1.12	4	2.08

300	18.99	±	1.10	3	20.45	±	1.63	3	1.38
301	19.11	±	0.62	4	20.55	±	0.37	4	0.55
302	18.42	±	2.03	3	21.96	±	0.53	4	2.11
303	18.18	±	1.11	3	19.85	±	0.29	4	0.31
304	17.87	±	0.89	4	22.21	±	0.66	4	2.26
306	18.47	±	0.80	4	23.96	±	1.66	3	3.85
307	18.71	±	0.67	4	22.84	±	0.97	4	2.29
308	20.33	±	1.70	4	22.80	±	1.91	4	0.60
309	19.46	±	1.80	4	23.19	±	3.56	4	2.48
310	17.92	±	0.40	3	22.73	±	1.40	4	0.70
311	20.39	±	0.88	3	22.21	±	1.66	3	1.20
313	19.27	±	0.41	4	21.26	±	1.14	4	0.68
315	19.53	±	0.75	4	21.56	±	0.56	4	1.03
316	19.39	±	1.22	4	23.86	±	1.49	4	1.26
318	18.95	±	0.58	4	23.25	±	1.01	4	1.23
320	19.24	±	1.21	4	22.58	±	0.75	4	1.35
323	21.26	±	1.65	4	22.50	±	2.53	3	1.11
324	18.02	±	0.57	4	21.27	±	2.44	3	1.28
325	19.04	±	0.95	4	20.54	±	1.87	3	1.00
327	18.86	±	1.62	3	25.45	±	1.33	4	2.32
329	21.60	±	1.51	4	21.24	±	0.44	3	0.10
331	18.82	±	0.91	3	20.51	±	1.00	4	1.60
332	20.26	±	1.24	4	21.19	±	1.18	4	1.04
333	18.47	±	1.25	3	21.31	±	1.57	3	0.86
334	20.12	±	1.86	4	21.90	±	1.25	4	1.34
337	20.01	±	0.93	3	22.01	±	1.47	3	0.76
338	19.59	±	1.79	4	23.43	±	2.84	4	2.43
339	18.87	±	1.17	4	24.35	±	1.32	4	1.71
342	22.76	±	2.36	3	21.24	±	2.08	3	-0.34
344	20.55	±	1.42	4	23.26	±	1.38	4	1.38
345	19.10	±	1.25	4	21.38	±	1.35	4	0.41
346	20.05	±	0.34	4	22.76	±	0.89	4	1.77
347	20.50	±	0.96	3	20.13	±	0.97	4	0.24

348	19.33	±	0.92	4	20.43	±	0.74	4	0.06
349	19.18	±	1.06	3	21.62	±	1.17	4	0.61
350	21.13	±	1.72	4	20.53	±	0.80	4	-0.19
352	19.15	±	1.03	3	24.31	±	1.50	4	2.50
355	18.69	±	0.55	4	21.29	±	1.32	4	1.84
356	21.29	±	0.47	4	22.93	±	1.20	3	0.27
357	19.76	±	0.88	4	19.57	±	0.46	4	0.03
358	18.65	±	0.37	4	23.13	±	2.18	3	1.72
359	18.51	±	0.60	4	21.05	±	1.06	4	0.67
361	19.83	±	0.66	4	22.63	±	0.51	4	0.55
363	19.99	±	0.56	4	22.68	±	0.70	4	1.66
364	19.64	±	0.61	3	24.06	±	1.38	4	2.36
367	20.62	±	0.91	4	20.74	±	1.14	4	-0.07
368	18.44	±	1.52	4	21.07	±	0.65	4	0.64
372	20.95	±	2.00	3	21.11	±	0.94	4	0.64
373	20.34	±	0.83	4	20.65	±	2.40	4	-0.31
374	19.34	±	0.73	4	20.80	±	1.93	3	1.49
375	20.91	±	1.93	4	21.95	±	1.29	4	1.63
377	19.79	±	1.38	4	21.41	±	0.82	4	0.98
378	19.41	±	2.52	3	21.88	±	1.53	3	0.80
379	18.53	±	1.00	4	22.69	±	0.41	4	2.11
381	18.95	±	0.77	4	22.81	±	2.06	4	1.73
382	20.52	±	0.96	3	22.63	±	1.14	3	1.08
383	18.81	±	0.62	4	21.58	±	1.30	4	1.27
385	19.82	±	0.52	3	19.65	±	0.87	4	1.37
386	19.76	±	1.31	4	21.17	±	2.49	3	-0.26
387	18.17	±	0.53	3	22.05	±	2.19	3	2.98
388	20.97	±	1.06	3	22.17	±	1.27	3	0.55
390	20.60	±	1.70	4	20.17	±	0.85	4	0.11
391	18.72	±	0.79	3	21.13	±	2.33	4	0.88
392	20.27	±	1.78	3	24.17	±	1.15	4	0.35
394	17.93	±	0.68	4	20.32	±	1.37	4	2.41
395	18.41	±	0.75	4	21.49	±	1.72	4	0.75

397	19.45	±	1.06	4	22.51	±	1.06	3	1.24
398	20.93	±	0.69	4	20.80	±	1.42	4	-0.11
399	21.09	±	1.83	3	23.49	±	1.07	4	1.75
400	19.61	±	1.38	4	22.00	±	1.68	4	0.41
401	17.82	±	1.29	3	20.43	±	0.48	4	1.93
402	18.98	±	0.69	4	23.35	±	3.21	3	1.30
403	19.06	±	1.06	3	22.07	±	1.52	3	2.12
404	19.22	±	0.34	4	21.23	±	0.85	4	0.99
405	18.21	±	0.72	4	21.85	±	1.13	4	1.16
406	20.88	±	1.28	4	22.79	±	1.33	4	-0.23
407	18.67	±	0.77	4	21.53	±	1.70	3	1.22
409	20.36	±	1.69	4	22.09	±	1.89	3	0.99
412	18.54	±	0.46	4	21.49	±	0.58	4	1.07
414	19.79	±	0.90	3	21.36	±	1.11	4	1.18
415	22.31	±	1.02	4	20.80	±	1.23	4	0.76
416	20.20	±	0.80	3	22.26	±	1.16	4	2.06
418	19.42	±	1.58	3	23.11	±	1.41	3	1.57
419	20.05	±	1.09	4	23.41	±	1.14	4	2.10
421	19.02	±	0.59	3	21.61	±	1.26	4	1.26
424	19.06	±	1.32	4	21.21	±	1.49	4	1.09
425	18.57	±	0.83	4	23.06	±	0.97	4	0.98
426	20.86	±	1.88	4	21.54	±	1.21	3	0.72
427	18.62	±	0.34	4	20.21	±	1.38	3	0.88
428	19.50	±	1.08	4	24.43	±	1.33	4	2.21
429	19.65	±	1.12	4	23.48	±	1.80	4	0.88
430	19.96	±	0.74	4	20.63	±	1.19	4	1.48
431	18.90	±	0.92	4	21.32	±	0.55	3	1.44
432	19.01	±	1.28	4	21.41	±	0.92	4	1.51
434	20.88	±	1.78	3	21.83	±	2.33	3	1.61
435	19.44	±	0.33	4	22.36	±	0.08	3	-0.16
436	20.55	±	0.53	4	20.96	±	0.63	4	0.56
437	18.92	±	0.88	3	22.59	±	0.37	3	1.59
439	19.70	±	0.84	4	20.79	±	0.57	4	-0.14

444	22.16	±	1.08	4	23.97	±	0.80	3	0.62
445	19.06	±	0.64	4	22.94	±	1.42	4	1.24
447	19.43	±	1.34	4	21.53	±	0.94	4	0.81
448	20.24	±	1.00	4	19.14	±	0.70	4	-0.48
450	18.83	±	0.66	4	22.68	±	0.93	4	1.29
451	17.70	±	0.70	4	22.65	±	0.81	4	1.58
452	18.34	±	0.92	3	22.56	±	2.16	4	1.93
453	17.04	±	1.04	3	21.27	±	2.01	3	1.86
454	21.09	±	2.86	4	22.27	±	0.57	3	1.30
455	20.24	±	1.70	4	22.87	±	1.43	4	0.68
459	19.66	±	1.15	3	21.41	±	0.62	3	0.58
460	20.58	±	0.33	3	20.30	±	0.61	3	0.01
461	20.37	±	1.40	3	21.95	±	2.16	3	1.85
462	19.37	±	0.67	3	21.58	±	1.20	4	1.72
463	19.68	±	1.41	3	23.01	±	0.68	3	0.60
464	18.97	±	1.01	4	19.51	±	1.32	4	0.40
465	21.04	±	1.33	4	20.36	±	0.10	4	-0.16
466	19.37	±	1.83	4	22.84	±	0.89	3	0.63
468	19.75	±	1.70	3	22.92	±	0.80	4	0.79
469	19.26	±	1.87	3	23.55	±	1.65	4	1.87
470	18.04	±	0.84	4	21.69	±	1.77	4	1.30
471	18.31	±	1.46	3	20.92	±	0.29	4	0.44
476	19.88	±	0.31	3	21.65	±	0.59	4	-0.17
477	18.20	±	1.33	3	23.64	±	0.77	3	2.20
478	18.46	±	0.33	3	19.69	±	0.94	3	1.73
479	18.18	±	0.42	4	21.51	±	1.58	4	1.18
480	20.30	±	1.02	3	20.03	±	0.73	4	0.07
481	16.95	±	0.50	4	20.99	±	1.05	4	2.17
483	19.26	±	0.82	3	23.64	±	0.78	3	0.46
484	16.72	±	0.53	3	22.35	±	0.12	3	2.77
485	20.72	±	0.92	3	22.35	±	1.95	4	1.75
486	18.64	±	0.86	4	20.08	±	1.90	3	0.05
489	19.53	±	1.82	3	25.35	±	1.56	4	2.71

490	18.60	±	0.60	4	20.38	±	0.63	3	0.93
493	20.02	±	1.06	3	19.12	±	0.65	4	0.26
494	20.87	±	1.25	4	22.52	±	0.33	4	1.92
497	20.98	±	1.45	3	22.24	±	0.50	4	0.14
498	19.94	±	1.46	3	20.76	±	1.61	4	0.96
499	18.68	±	1.32	3	22.07	±	1.98	3	1.29
500	20.94	±	0.89	4	22.14	±	1.09	4	0.46
502	18.27	±	0.48	3	20.56	±	0.95	4	1.94
503	19.56	±	1.87	4	20.05	±	0.79	4	0.67
504	21.00	±	2.06	3	23.95	±	0.57	3	1.26
505	19.29	±	1.14	4	24.11	±	0.82	3	2.39
506	20.22	±	1.41	4	19.15	±	0.59	4	-0.56
507	19.29	±	0.95	4	23.76	±	2.22	4	1.52
514	17.80	±	0.32	4	18.94	±	1.40	4	-0.22
515	20.49	±	1.43	4	22.34	±	1.27	4	1.46
516	19.08	±	1.36	3	20.20	±	1.97	4	1.33
520	19.88	±	0.57	4	23.46	±	1.74	4	0.73
523	20.90	±	1.17	4	20.60	±	1.21	4	-1.16
524	18.74	±	0.70	4	21.05	±	1.30	4	2.16
525	21.77	±	0.97	4	20.25	±	0.66	4	-0.52
527	18.40	±	1.03	3	21.48	±	1.00	3	0.55
528	18.97	±	0.95	3	21.03	±	1.03	4	1.20
529	21.54	±	1.47	4	21.42	±	0.31	4	-0.20
531	19.04	±	0.82	4	19.56	±	0.86	4	0.54
532	22.67	±	1.43	4	25.54	±	2.03	3	0.39
534	18.18	±	0.23	4	22.80	±	0.83	4	3.41
535	16.62	±	0.34	4	23.57	±	1.08	4	3.48
536	20.21	±	0.69	3	22.57	±	2.15	3	-0.05
537	17.99	±	0.49	4	20.59	±	1.28	3	0.47
541	19.60	±	0.29	3	20.90	±	0.41	3	-0.03
543	19.92	±	1.68	3	20.42	±	0.57	3	0.69
545	18.80	±	1.54	3	23.42	±	0.97	4	2.27
546	20.53	±	0.67	4	22.24	±	1.49	4	1.30

548	19.19	±	1.12	3	24.69	±	1.64	4	2.24
549	20.47	±	1.46	4	21.99	±	2.29	4	0.30
550	19.38	±	0.82	4	21.27	±	0.60	4	1.58
552	19.43	±	0.06	3	21.60	±	1.24	3	1.11
553	19.46	±	0.87	4	20.36	±	0.76	4	1.31
555	20.31	±	1.47	4	20.70	±	1.43	4	0.90
556	20.00	±	1.74	4	21.59	±	1.87	4	1.56
557	19.19	±	1.61	3	20.65	±	1.10	4	0.19
558	20.19	±	2.77	3	22.46	±	2.75	3	0.71
559	21.06	±	0.70	3	23.51	±	1.52	4	0.99
561	19.98	±	1.41	4	22.06	±	1.96	4	1.16
562	18.23	±	0.54	4	20.96	±	1.70	4	1.55
563	17.60	±	0.56	3	24.62	±	1.07	4	3.35
564	21.98	±	0.73	3	21.47	±	1.37	4	-0.01
565	20.35	±	0.66	4	21.66	±	1.17	3	0.16
570	19.18	±	0.25	4	20.30	±	0.22	4	-0.19
571	20.71	±	1.27	4	19.74	±	1.23	4	0.27
572	20.52	±	1.08	4	20.91	±	0.92	4	-0.22
574	19.84	±	0.69	3	21.84	±	0.98	4	0.46
575	18.56	±	0.38	4	21.18	±	1.47	4	0.99
576	18.19	±	0.73	3	22.63	±	1.86	4	2.16
578	18.92	±	0.70	4	21.68	±	2.21	4	1.29
579	19.98	±	1.48	4	20.05	±	0.88	4	0.75
580	22.24	±	0.35	3	20.38	±	1.12	4	0.14
581	19.50	±	2.20	3	22.29	±	1.63	4	1.66
582	17.86	±	0.61	3	20.54	±	3.20	3	1.48
583	20.08	±	1.98	4	20.10	±	0.40	4	1.12
584	17.68	±	0.48	3	21.00	±	0.50	4	2.67
585	18.84	±	0.89	3	20.96	±	1.26	4	0.56
586	21.00	±	2.58	3	20.39	±	1.81	4	0.32
587	20.45	±	0.87	3	20.81	±	1.23	3	-0.21
588	18.47	±	0.50	4	22.52	±	0.85	4	1.30
589	21.18	±	1.45	3	20.73	±	0.65	4	0.61

590	18.22	±	0.44	4	21.05	±	0.29	3	1.19
591	18.96	±	0.76	4	19.97	±	0.55	3	0.47
592	21.11	±	1.23	3	21.99	±	0.84	4	0.18
593	20.64	±	1.49	3	22.12	±	0.75	2	2.28
594	19.69	±	1.90	4	19.87	±	2.70	3	-0.04
596	19.21	±	1.46	3	21.79	±	1.47	4	0.84
599	19.77	±	0.70	3	21.36	±	1.71	4	0.62
600	17.69	±	0.35	4	22.58	±	1.37	4	2.70
602	21.72	±	0.76	4	19.52	±	1.25	4	0.61
603	18.34	±	1.07	3	20.26	±	0.38	4	1.43
604	20.32	±	0.68	3	22.99	±	1.12	4	0.61
605	18.93	±	0.19	3	18.97	±	1.02	3	0.64
606	19.22	±	1.14	4	23.62	±	1.28	3	0.31
608	20.13	±	0.38	4	22.57	±	1.45	3	0.26
609	19.36	±	1.75	4	26.01	±	2.02	4	3.97
610	20.02	±	1.93	3	20.84	±	0.56	4	0.86
611	20.36	±	0.31	4	21.40	±	0.96	3	-0.65
612	17.71	±	0.93	4	21.17	±	0.70	3	1.24
613	19.27	±	0.97	3	20.48	±	1.40	4	0.60
614	18.71	±	1.11	4	22.62	±	1.32	4	0.22
615	19.98	±	0.98	4	24.84	±	1.15	4	3.99
617	19.64	±	1.18	4	22.29	±	0.65	4	1.95
619	20.37	±	1.44	4	20.50	±	1.71	4	-0.06
620	20.59	±	1.31	3	19.37	±	1.12	4	-0.65
622	18.77	±	0.75	4	19.07	±	1.23	3	0.84
623	20.45	±	1.24	4	21.09	±	1.22	4	0.68
624	18.15	±	0.55	4	20.63	±	1.00	4	0.09
625	19.17	±	0.79	3	21.54	±	1.06	4	0.78
626	21.87	±	1.47	4	21.34	±	1.69	4	1.02
627	20.78	±	1.17	4	20.32	±	0.81	4	-1.17
628	18.83	±	0.89	4	20.04	±	1.45	3	0.74
629	16.97	±	0.46	4	21.07	±	1.55	4	1.05
630	17.32	±	1.11	3	22.88	±	0.78	4	2.78

631	20.05	±	2.04	4	21.26	±	1.74	4	-0.06
632	19.26	±	0.92	4	20.94	±	1.21	4	1.51
633	18.33	±	0.63	3	20.97	±	0.27	4	1.05
634	19.58	±	0.42	4	22.90	±	1.48	3	0.97
635	18.07	±	0.31	4	19.68	±	0.78	4	2.31
636	18.85	±	0.75	4	22.46	±	0.70	3	1.58
637	18.67	±	0.93	4	21.30	±	0.68	4	0.89
638	19.23	±	0.77	4	20.73	±	1.22	4	1.16
640	21.11	±	1.25	4	21.66	±	0.98	3	0.51
641	17.89	±	0.89	4	21.36	±	1.70	4	1.77
642	18.49	±	0.42	4	21.15	±	1.02	4	0.31
643	19.75	±	1.34	4	20.37	±	0.79	4	0.17
644	19.37	±	0.67	4	22.41	±	2.19	4	0.85
645	19.85	±	1.92	3	21.24	±	1.71	4	1.92
646	17.78	±	0.99	3	20.95	±	1.55	3	1.95
647	17.71	±	0.70	4	19.15	±	0.70	3	0.94
648	18.60	±	0.35	3	21.91	±	1.69	3	2.21
649	17.74	±	1.05	4	22.55	±	1.14	4	3.61
650	17.66	±	0.78	3	23.76	±	1.26	4	2.66
651	20.72	±	2.51	3	21.71	±	2.27	3	1.12
652	17.18	±	0.43	3	24.03	±	0.43	3	1.13
653	18.22	±	1.03	3	21.28	±	0.94	4	1.74
654	21.24	±	1.55	4	18.52	±	0.49	3	-0.31
655	17.32	±	0.43	4	24.70	±	1.83	3	3.28
656	17.91	±	1.30	3	20.52	±	1.35	4	1.08
657	19.59	±	0.98	3	23.16	±	0.90	4	1.27
658	18.06	±	0.77	3	21.49	±	0.48	3	1.04
659	18.86	±	0.75	4	24.31	±	0.73	3	2.64
660	18.66	±	0.87	4	23.02	±	1.41	4	2.48
661	19.14	±	0.95	4	24.15	±	1.48	4	3.16
662	20.06	±	0.94	4	22.14	±	1.24	4	1.48
663	19.31	±	1.59	4	23.26	±	0.85	4	1.41
665	18.22	±	0.67	4	21.15	±	1.44	3	0.16

667	19.62	±	0.77	4	22.84	±	1.70	4	0.78
668	17.31	±	0.77	3	20.50	±	1.51	4	2.00
670	18.35	±	1.06	4	20.42	±	0.82	4	-0.20
671	22.42	±	1.09	4	22.25	±	1.75	4	-0.17
673	21.90	±	1.98	3	22.33	±	0.82	3	0.64
674	20.33	±	2.17	3	21.75	±	0.92	4	0.28
677	20.10	±	1.63	3	22.68	±	1.45	4	1.65
678	18.75	±	0.71	4	20.40	±	1.07	4	2.20
680	19.14	±	1.08	4	19.98	±	1.53	3	-0.27
682	19.15	±	0.41	4	21.89	±	1.52	3	1.27
683	17.58	±	0.80	3	23.72	±	2.13	4	2.42
684	18.38	±	0.37	4	20.94	±	1.14	4	1.65
685	19.94	±	1.52	3	19.52	±	1.26	4	-0.06
686	19.82	±	1.34	4	19.78	±	0.76	4	-0.21
687	18.84	±	0.84	3	22.03	±	1.43	4	2.00
688	19.91	±	0.91	3	21.88	±	2.21	3	1.94
690	18.90	±	0.98	3	21.24	±	1.19	4	0.96
58-861	19.83	±	0.67	14	20.88	±	0.45	16	1.25
P-36	19.30	±	0.47	16	21.27	±	0.83	13	-0.66
P-64	19.12	±	0.36	16	20.83	±	0.63	16	0.60
Poli	19.64	±	0.64	13	21.82	±	0.75	15	1.52

Supplementary Table S2| Genotypic mean canopy temperature (T_c, °C) and standard error (SE, °C) for WW and mDr treatments. Data are based on Matlab segmentation. Values are computed based on the reported number of replicates (rep.). Stress Susceptibility Index (SSI) is also presented for each genotype.

	WW				mDr				
**genotype**	**T_c**		***SE***	**rep.**	**T_c**		***SE***	**rep.**	**SSI**
1	21.40	±	0.76	4	21.80	±	1.74	4	0.19
2	18.50	±	0.89	4	22.59	±	1.04	3	2.18
3	21.71	±	0.98	3	20.36	±	1.01	3	-0.62
4	21.43	±	1.35	4	22.25	±	1.27	3	0.35
6	20.03	±	1.42	3	22.26	±	0.82	4	1.17
7	17.78	±	0.69	3	21.66	±	1.59	4	2.28
8	20.22	±	0.40	4	21.46	±	0.78	4	0.60
10	19.99	±	0.79	4	22.10	±	1.12	4	1.04
13	19.25	±	1.11	4	21.07	±	1.06	3	0.84
15	20.12	±	1.62	4	22.86	±	0.68	4	1.34
16	20.12	±	1.49	4	22.85	±	0.96	4	1.33
17	18.64	±	0.91	4	19.95	±	1.57	4	0.69
20	18.59	±	0.59	4	22.62	±	1.55	4	2.13
22	22.81	±	0.35	4	21.34	±	0.67	4	-0.63
25	17.81	±	0.78	4	20.28	±	0.35	4	1.36
26	20.90	±	1.54	3	22.29	±	2.37	3	0.52
28	20.34	±	1.52	3	22.41	±	1.85	4	0.94
29	19.77	±	0.91	4	20.72	±	0.27	4	0.47
30	22.22	±	1.24	4	21.85	±	0.58	4	-0.16
32	20.11	±	1.98	4	21.49	±	0.52	4	0.67
33	21.18	±	1.45	3	20.91	±	1.33	4	-0.09
34	19.25	±	0.49	4	21.01	±	1.58	4	0.89
35	18.56	±	0.99	3	22.53	±	1.42	4	1.95
36	17.53	±	0.70	3	20.44	±	1.12	3	1.53
37	22.48	±	1.49	3	23.24	±	1.14	4	0.40
38	19.83	±	1.12	3	21.78	±	2.07	3	0.85
39	19.73	±	1.03	4	20.93	±	0.56	3	0.52

40	20.71 ± 0.25	3	20.83 ± 1.44	4	0.15
42	20.46 ± 0.62	3	23.00 ± 1.35	3	1.25
43	20.40 ± 1.09	3	22.92 ± 0.77	4	1.32
45	17.54 ± 0.40	3	22.67 ± 1.12	4	2.78
46	18.27 ± 0.93	4	21.36 ± 1.84	4	1.66
48	19.13 ± 1.34	3	19.65 ± 2.20	3	0.38
49	19.91 ± 1.18	4	20.80 ± 1.24	4	0.44
50	18.64 ± 1.20	3	22.45 ± 0.79	4	1.86
51	18.27 ± 0.55	4	21.91 ± 1.11	4	1.95
52	20.66 ± 1.09	3	23.21 ± 0.62	4	1.32
53	20.30 ± 0.99	4	22.97 ± 1.12	4	1.29
54	19.30 ± 0.98	4	20.85 ± 1.07	4	0.79
55	19.05 ± 1.54	3	20.65 ± 0.29	3	0.71
56	21.48 ± 0.91	4	20.65 ± 2.88	3	-0.37
57	17.68 ± 0.26	4	21.77 ± 1.52	3	2.37
58	19.94 ± 0.61	4	20.98 ± 0.90	3	0.52
59	19.68 ± 1.70	4	20.99 ± 0.76	4	0.65
61	20.21 ± 1.27	4	20.87 ± 1.41	4	0.32
63	20.19 ± 1.40	4	20.56 ± 1.71	4	0.18
65	21.28 ± 0.74	4	20.62 ± 0.58	4	-0.30
66	19.60 ± 0.28	4	21.15 ± 0.75	4	0.78
67	20.69 ± 0.66	4	21.77 ± 1.63	4	0.51
68	19.17 ± 0.85	4	22.20 ± 0.95	4	1.55
71	18.64 ± 1.34	3	20.62 ± 0.60	4	0.97
73	19.63 ± 0.59	4	22.22 ± 1.48	4	1.30
75	20.05 ± 0.94	4	22.12 ± 1.48	3	1.02
76	19.99 ± 1.14	4	21.11 ± 0.82	4	0.55
77	17.86 ± 0.93	3	20.75 ± 1.33	4	1.68
78	18.83 ± 1.00	3	22.80 ± 0.95	4	2.16
79	21.79 ± 1.41	3	22.30 ± 0.62	4	0.30
80	19.48 ± 1.32	4	20.80 ± 1.64	4	0.67
81	19.33 ± 1.60	3	22.34 ± 0.54	3	1.65
82	17.39 ± 0.41	4	19.63 ± 0.71	4	1.26

83	19.10 ± 1.54	3	21.22 ± 0.75	3	1.21
84	19.39 ± 1.01	4	21.66 ± 1.78	4	1.15
85	21.29 ± 0.70	4	20.81 ± 1.51	4	-0.22
87	17.84 ± 0.92	3	20.35 ± 1.46	3	1.43
88	20.97 ± 1.50	3	22.52 ± 1.49	4	0.80
91	19.00 ± 0.72	4	23.33 ± 1.73	4	2.23
92	20.51 ± 1.36	4	19.65 ± 0.34	3	-0.43
93	19.85 ± 2.15	4	20.56 ± 1.44	4	0.35
94	18.25 ± 0.92	3	21.30 ± 0.84	3	1.54
97	19.99 ± 1.65	3	19.19 ± 1.20	3	-0.47
98	21.02 ± 1.91	4	21.06 ± 0.78	4	0.02
100	21.12 ± 1.18	4	20.24 ± 1.75	4	-0.41
101	19.54 ± 0.90	4	20.84 ± 0.81	4	0.65
104	19.96 ± 1.60	4	21.40 ± 1.55	4	0.71
106	19.17 ± 0.50	4	21.98 ± 1.73	4	1.43
107	19.13 ± 0.96	4	22.54 ± 0.62	4	1.75
108	17.91 ± 0.59	3	19.44 ± 0.29	4	0.70
110	18.09 ± 0.62	3	20.74 ± 0.42	3	1.30
111	18.45 ± 0.48	4	20.97 ± 1.07	4	1.34
112	19.08 ± 0.69	3	20.35 ± 0.83	4	0.76
114	18.97 ± 1.35	3	21.97 ± 0.49	3	1.65
116	18.86 ± 1.39	4	20.03 ± 0.77	4	0.61
117	20.80 ± 1.78	3	19.77 ± 0.95	3	-0.42
118	18.22 ± 0.87	4	23.31 ± 0.59	3	2.83
119	20.53 ± 1.83	3	19.40 ± 1.18	3	-0.63
120	20.46 ± 0.80	4	19.52 ± 0.76	4	-0.45
122	20.21 ± 2.09	3	21.47 ± 1.55	4	0.55
124	18.54 ± 1.33	4	21.29 ± 1.49	4	1.46
126	20.63 ± 0.91	4	21.88 ± 1.17	4	0.59
127	18.58 ± 0.71	4	20.49 ± 1.10	3	0.93
128	20.50 ± 1.83	4	20.80 ± 1.25	4	0.14
129	20.17 ± 1.40	4	21.75 ± 0.73	4	0.77
130	21.53 ± 1.89	3	22.91 ± 2.20	4	0.57

131	19.48	±	0.90	5	22.22	±	1.64	4	1.31
133	18.80	±	1.42	4	23.44	±	1.82	4	2.41
135	18.54	±	1.41	3	20.87	±	0.81	4	1.36
136	19.95	±	0.95	3	18.82	±	0.93	3	-0.54
138	19.38	±	1.54	4	22.03	±	0.52	3	1.26
139	20.15	±	1.66	3	22.21	±	1.58	4	1.11
141	20.54	±	1.77	3	20.94	±	0.71	4	0.26
142	19.31	±	0.99	4	21.94	±	0.66	4	1.33
144	18.86	±	1.30	3	22.00	±	1.87	3	1.42
145	17.84	±	0.61	3	21.17	±	1.67	4	1.96
146	17.94	±	1.14	4	19.26	±	1.14	4	0.72
148	20.04	±	2.36	3	21.70	±	0.95	4	0.69
150	19.32	±	1.60	3	20.52	±	1.66	4	0.55
151	18.52	±	0.79	3	23.83	±	0.69	3	2.88
152	19.57	±	0.57	4	20.48	±	0.63	4	0.45
153	21.16	±	1.92	3	20.33	±	1.03	3	-0.42
154	18.32	±	1.61	3	21.91	±	1.39	4	1.85
155	17.16	±	0.49	3	21.16	±	1.41	4	2.20
156	21.85	±	1.79	3	21.42	±	1.46	4	-0.13
158	20.55	±	1.10	3	20.29	±	0.93	4	-0.06
159	18.66	±	0.43	3	22.36	±	1.98	3	2.05
160	19.01	±	0.58	4	22.22	±	0.86	3	1.66
161	19.94	±	1.38	4	21.31	±	1.83	3	0.68
163	23.06	±	1.67	4	18.14	±	0.69	3	-2.08
164	18.37	±	0.93	4	21.79	±	1.75	4	1.83
165	21.17	±	1.18	3	22.64	±	1.48	4	0.57
166	20.28	±	2.46	3	20.31	±	0.46	4	-0.04
167	19.23	±	0.97	4	23.68	±	0.81	4	2.27
168	19.57	±	0.84	3	21.14	±	0.84	4	0.66
169	17.20	±	0.45	3	20.54	±	2.03	3	1.85
170	18.60	±	1.25	4	20.75	±	0.91	4	1.13
171	22.51	±	1.49	3	23.17	±	0.82	4	0.24
172	19.64	±	1.42	4	20.05	±	1.43	4	0.20

173	18.78	±	1.44	4	20.13	±	0.72	3	0.80
175	19.96	±	1.79	3	20.82	±	0.66	4	0.36
176	21.83	±	1.78	4	20.64	±	0.76	4	-0.54
177	19.18	±	1.92	3	22.03	±	0.97	3	1.55
178	22.72	±	1.93	3	22.21	±	2.17	4	-0.13
179	20.94	±	0.44	3	20.17	±	1.46	3	-0.29
181	19.00	±	0.92	4	20.40	±	1.48	3	0.70
182	20.00	±	0.50	4	21.51	±	1.10	4	0.74
183	17.56	±	0.46	3	20.98	±	1.52	4	2.05
184	18.69	±	1.19	3	24.87	±	1.65	3	3.08
185	17.95	±	0.79	4	24.17	±	1.94	4	3.40
187	20.11	±	1.79	4	23.96	±	1.02	4	1.87
188	19.37	±	1.72	3	22.27	±	0.68	4	1.40
189	18.11	±	0.59	4	20.74	±	1.97	4	1.43
190	18.56	±	1.44	3	21.89	±	0.75	4	1.69
191	18.75	±	0.68	4	20.65	±	2.04	4	0.99
193	18.82	±	1.16	4	22.56	±	1.72	4	1.95
195	20.53	±	1.81	3	22.72	±	1.70	4	0.92
196	17.77	±	0.41	4	21.60	±	0.97	4	2.12
198	20.91	±	1.83	4	21.76	±	1.24	4	0.40
199	22.04	±	1.40	3	22.62	±	2.09	4	0.35
200	22.38	±	1.48	4	20.03	±	0.32	3	-1.05
201	20.32	±	1.07	4	23.00	±	0.65	4	1.29
202	19.95	±	1.63	4	20.19	±	1.69	3	0.10
203	18.96	±	1.00	4	21.80	±	1.03	4	1.47
205	19.28	±	0.77	4	21.87	±	1.59	3	1.33
206	19.83	±	1.04	3	22.39	±	1.67	4	1.20
207	20.28	±	1.29	4	19.50	±	0.84	4	-0.38
208	19.84	±	1.21	3	23.06	±	1.11	3	1.63
209	18.77	±	0.64	4	20.24	±	0.87	3	0.75
210	17.31	±	0.61	4	24.07	±	1.78	3	3.74
214	19.97	±	0.87	3	21.40	±	1.36	3	0.59
215	19.66	±	1.81	4	20.79	±	1.33	3	0.65

216	19.85	±	1.72	3	22.34	±	1.20	3	1.15
218	21.08	±	1.19	4	21.10	±	2.09	4	0.01
219	22.26	±	1.25	3	19.90	±	1.54	3	-1.11
220	18.23	±	0.97	4	20.52	±	1.34	4	1.23
221	21.01	±	1.98	4	22.72	±	0.58	4	0.80
222	19.33	±	0.97	4	18.69	±	0.48	4	-0.32
223	20.43	±	0.95	3	23.02	±	0.92	4	1.18
224	17.58	±	0.93	3	20.68	±	0.46	4	1.65
225	20.04	±	1.70	3	20.99	±	1.26	3	0.39
226	20.85	±	1.18	3	21.36	±	1.72	4	0.13
231	18.77	±	1.61	4	23.80	±	1.16	4	2.63
232	18.99	±	0.59	4	20.12	±	0.63	3	0.68
233	19.48	±	1.94	3	20.38	±	0.76	4	0.53
234	18.98	±	0.91	3	23.28	±	0.62	4	2.35
235	18.56	±	2.37	3	22.17	±	0.39	4	1.77
237	22.17	±	1.60	4	22.07	±	1.77	4	-0.04
238	21.23	±	2.65	3	21.53	±	1.16	3	0.31
239	20.43	±	1.84	4	24.45	±	0.79	3	1.91
240	20.09	±	1.29	4	22.01	±	2.85	3	0.94
241	19.18	±	0.96	4	22.09	±	0.42	4	1.48
242	19.85	±	1.77	3	21.35	±	0.40	3	0.69
243	18.89	±	1.21	4	21.77	±	1.09	4	1.49
244	17.86	±	0.65	3	20.51	±	0.79	4	1.58
245	18.59	±	0.66	4	22.01	±	1.07	4	1.81
247	20.52	±	0.63	3	19.06	±	0.84	4	-0.61
248	19.73	±	3.24	3	22.90	±	0.91	4	1.45
251	20.72	±	1.32	4	21.52	±	1.18	3	0.31
252	20.88	±	1.54	4	19.67	±	0.59	3	-0.64
253	20.45	±	0.75	4	20.05	±	1.16	4	-0.19
254	18.11	±	0.98	3	20.21	±	0.83	3	0.92
255	19.24	±	1.20	4	21.29	±	0.54	4	1.05
256	22.57	±	1.86	4	23.17	±	0.79	4	0.26
257	22.12	±	1.17	4	21.60	±	1.22	4	-0.23

258	19.16	±	0.68	4	22.90	±	0.51	4	1.91
259	19.66	±	0.44	4	22.36	±	1.30	3	1.33
260	19.94	±	0.43	3	21.08	±	1.67	4	0.63
261	20.38	±	1.18	4	20.77	±	2.05	4	0.18
264	20.90	±	0.87	4	21.78	±	0.53	4	0.42
265	22.29	±	1.92	4	22.03	±	1.01	4	-0.11
266	17.96	±	0.52	4	19.28	±	0.38	4	0.72
267	20.56	±	1.19	4	21.09	±	0.25	3	0.34
268	21.00	±	1.57	4	20.51	±	0.55	3	-0.25
271	19.87	±	1.49	4	23.90	±	1.03	4	1.99
273	19.57	±	0.91	3	22.51	±	1.29	4	1.34
275	17.93	±	1.17	4	19.93	±	0.76	3	1.01
276	20.10	±	1.19	3	21.73	±	1.85	4	0.90
278	22.12	±	1.77	3	22.30	±	1.11	4	0.03
280	20.64	±	1.43	4	20.10	±	0.68	4	-0.26
281	19.75	±	1.14	4	20.54	±	0.94	4	0.39
282	18.59	±	1.48	3	22.11	±	1.58	4	1.78
283	19.78	±	0.70	4	19.20	±	1.46	3	-0.20
287	17.54	±	0.74	3	22.57	±	1.88	4	2.73
288	18.51	±	0.56	3	21.34	±	1.98	4	1.59
289	20.03	±	1.31	3	19.65	±	0.90	4	-0.09
290	19.12	±	1.51	3	20.39	±	1.64	4	0.76
292	20.28	±	2.05	4	22.91	±	1.19	4	1.27
293	17.78	±	0.52	4	21.95	±	0.68	3	2.40
295	19.85	±	1.54	4	20.58	±	1.57	3	0.45
296	18.69	±	0.37	3	19.58	±	0.62	4	0.34
297	20.62	±	0.59	4	21.30	±	1.01	4	0.32
298	18.91	±	1.52	3	19.97	±	0.21	4	0.66
299	20.51	±	0.82	3	23.93	±	1.02	4	1.57
300	18.99	±	1.10	3	20.44	±	1.60	3	0.78
301	19.16	±	0.64	4	20.56	±	0.38	4	0.71
302	18.48	±	2.00	3	21.88	±	0.48	4	1.89
303	18.18	±	1.11	3	19.85	±	0.29	4	0.83

304	17.88	±	0.88	4	22.21	±	0.65	4	2.37
306	18.47	±	0.80	4	23.95	±	1.66	3	2.92
307	18.67	±	0.63	4	22.85	±	0.72	4	2.19
308	20.17	±	1.53	4	22.46	±	1.56	4	1.11
309	19.61	±	1.87	4	21.82	±	2.27	4	1.10
310	17.92	±	0.40	3	22.66	±	1.35	4	2.52
311	20.39	±	0.88	3	22.12	±	1.62	3	0.91
313	19.37	±	0.44	4	21.29	±	1.17	4	0.97
315	19.87	±	1.08	4	21.57	±	0.60	4	0.84
316	19.39	±	1.22	4	24.18	±	1.31	4	2.42
318	18.95	±	0.58	4	23.19	±	1.07	4	2.19
320	19.33	±	1.25	4	22.62	±	0.84	4	1.67
323	20.83	±	1.24	4	21.72	±	2.12	3	0.51
324	18.02	±	0.57	4	21.26	±	2.42	3	1.77
325	19.08	±	0.97	4	20.56	±	1.87	3	0.69
327	18.87	±	1.61	3	24.68	±	1.10	4	3.15
329	21.63	±	1.44	4	21.28	±	0.45	3	-0.18
331	18.85	±	0.88	3	20.52	±	1.00	4	0.98
332	20.26	±	1.24	4	20.91	±	0.95	4	0.31
333	18.47	±	1.25	3	21.36	±	1.64	3	1.58
334	20.20	±	1.84	4	21.70	±	1.24	4	0.73
337	20.03	±	0.93	3	21.78	±	1.54	3	0.94
338	19.35	±	1.55	4	22.06	±	1.84	4	1.37
339	18.91	±	1.13	4	23.89	±	1.06	4	2.58
344	20.54	±	1.28	4	23.15	±	1.27	4	1.25
345	19.04	±	1.19	4	21.38	±	1.36	4	1.21
346	20.15	±	0.40	4	22.52	±	0.81	4	1.16
347	20.50	±	0.96	3	20.29	±	1.11	4	-0.03
348	19.33	±	0.92	4	20.55	±	0.83	4	0.62
349	19.18	±	1.06	3	21.51	±	1.09	4	1.06
350	20.69	±	1.30	4	20.67	±	0.77	4	-0.01
352	19.15	±	1.03	3	23.82	±	1.27	4	2.52
355	18.78	±	0.62	4	21.08	±	1.14	4	1.20

356	21.75	±	0.32	4	22.76	±	1.15	3	0.46
357	19.76	±	0.88	4	19.61	±	0.47	4	-0.07
358	18.75	±	0.43	4	22.85	±	1.94	3	2.12
359	18.72	±	0.81	4	20.89	±	1.03	4	1.13
361	19.90	±	0.67	4	22.63	±	0.51	4	1.34
363	20.02	±	0.58	4	22.41	±	0.77	4	1.17
364	19.64	±	0.61	3	23.70	±	1.28	4	2.11
367	20.62	±	0.91	4	20.73	±	1.12	4	0.05
368	18.40	±	1.41	4	21.06	±	0.65	4	1.42
372	20.68	±	1.95	3	21.29	±	0.90	4	0.18
373	20.53	±	0.85	4	20.54	±	2.30	4	0.01
374	19.34	±	0.73	4	20.48	±	1.74	3	0.58
375	21.00	±	1.77	4	21.97	±	1.31	4	0.45
377	19.79	±	1.38	4	21.49	±	0.84	4	0.84
378	19.54	±	2.64	3	21.79	±	1.40	3	1.13
379	18.53	±	1.00	4	22.46	±	0.22	4	2.08
381	18.95	±	0.77	4	22.66	±	2.02	4	1.92
382	20.72	±	1.00	3	22.50	±	1.23	3	0.65
383	18.81	±	0.62	4	21.57	±	1.31	4	1.44
385	19.82	±	0.52	3	19.67	±	0.87	4	-0.19
386	19.76	±	1.31	4	20.76	±	2.12	3	0.42
387	18.17	±	0.53	3	21.92	±	2.06	3	1.92
388	21.21	±	1.30	3	22.14	±	1.24	3	0.48
390	20.73	±	1.80	4	20.22	±	0.85	4	-0.24
391	18.72	±	0.79	3	20.90	±	2.15	4	1.07
392	20.34	±	1.80	3	24.00	±	1.19	4	1.63
394	17.93	±	0.68	4	20.32	±	1.36	4	1.30
395	18.41	±	0.75	4	21.32	±	1.53	4	1.55
397	19.54	±	1.11	4	22.04	±	0.78	3	1.34
398	21.46	±	1.11	4	20.65	±	1.29	4	-0.37
399	21.74	±	2.29	3	23.56	±	1.04	4	0.71
400	19.77	±	1.53	4	22.07	±	1.66	4	1.14
401	17.85	±	1.27	3	20.36	±	0.43	4	1.24

403	19.06	±	1.06	3	21.64	±	1.17	3	1.45
404	19.18	±	0.35	4	21.05	±	0.69	4	0.96
405	18.30	±	0.79	4	22.16	±	1.27	4	2.06
406	20.77	±	1.18	4	22.82	±	1.33	4	0.96
407	18.67	±	0.77	4	21.44	±	1.60	3	1.47
409	20.36	±	1.69	4	22.05	±	1.88	3	0.80
412	18.58	±	0.48	4	21.64	±	0.65	4	1.61
414	19.79	±	0.90	3	21.36	±	1.10	4	0.71
415	22.16	±	0.87	4	20.86	±	1.27	4	-0.58
416	20.52	±	0.80	3	22.17	±	1.14	4	0.86
418	19.42	±	1.58	3	23.06	±	1.38	3	1.94
419	20.05	±	1.09	4	23.31	±	1.18	4	1.60
421	19.02	±	0.59	3	21.64	±	1.23	4	1.29
424	19.06	±	1.32	4	21.19	±	1.49	4	1.10
425	18.57	±	0.83	4	22.82	±	0.79	4	2.24
426	20.70	±	1.74	4	21.54	±	1.23	3	0.48
427	18.68	±	0.33	4	20.22	±	1.39	3	0.90
428	19.50	±	1.08	4	24.05	±	1.24	4	2.28
429	19.65	±	1.12	4	23.19	±	1.63	4	1.77
430	19.98	±	0.73	4	20.56	±	1.12	4	0.29
431	18.90	±	0.92	4	21.33	±	0.56	3	1.35
432	19.22	±	1.47	4	21.18	±	0.78	4	1.00
434	20.87	±	1.77	3	21.98	±	2.39	3	0.57
435	19.19	±	0.13	4	22.07	±	0.35	3	1.39
436	20.55	±	0.53	4	21.00	±	0.64	4	0.22
437	18.92	±	0.88	3	22.53	±	0.37	3	2.01
439	19.87	±	0.87	4	20.79	±	0.57	4	0.45
444	22.25	±	1.17	4	23.59	±	0.90	3	0.52
445	19.06	±	0.64	4	22.83	±	1.31	4	1.94
447	19.33	±	1.27	4	21.62	±	0.94	4	1.16
448	20.37	±	1.04	4	19.32	±	0.79	4	-0.50
450	18.83	±	0.66	4	22.63	±	0.88	4	1.98
451	17.70	±	0.70	4	22.50	±	0.67	4	2.66

452	18.24	±	0.83	3	22.50	±	2.03	4	2.21
453	17.11	±	0.99	3	21.27	±	2.00	3	2.31
454	20.35	±	2.13	4	22.34	±	0.54	3	0.97
455	20.26	±	1.69	4	22.92	±	1.47	4	1.29
459	19.64	±	1.16	3	21.24	±	0.73	3	0.76
460	20.58	±	0.33	3	20.32	±	0.62	3	-0.08
461	20.42	±	1.45	3	21.60	±	1.86	3	0.72
462	19.37	±	0.67	3	21.58	±	1.20	4	0.99
463	19.59	±	1.32	3	22.95	±	0.76	3	1.89
464	18.97	±	1.01	4	19.44	±	1.28	4	0.24
465	21.44	±	1.55	4	20.56	±	0.29	4	-0.40
466	19.29	±	1.76	4	22.83	±	0.94	3	1.81
468	19.82	±	1.63	3	22.90	±	0.69	4	1.39
469	19.19	±	1.80	3	23.24	±	1.43	4	2.19
470	18.04	±	0.83	4	21.68	±	1.77	4	1.98
471	18.31	±	1.46	3	20.93	±	0.29	4	1.53
473	18.28	±	1.21	4	23.58	±	1.54	4	2.84
476	19.88	±	0.31	3	21.64	±	0.59	4	0.81
477	18.24	±	1.37	3	23.30	±	0.66	3	2.48
478	18.46	±	0.33	3	19.69	±	0.93	3	0.86
479	18.18	±	0.42	4	21.53	±	1.58	4	1.81
480	20.30	±	1.02	3	20.12	±	0.67	4	-0.02
481	17.04	±	0.41	4	21.15	±	1.17	4	2.36
483	19.26	±	0.82	3	23.40	±	0.90	3	2.12
484	16.77	±	0.49	3	21.97	±	0.50	3	2.96
485	20.72	±	0.92	3	22.20	±	1.80	4	0.80
486	18.64	±	0.86	4	20.08	±	1.90	3	0.68
489	19.66	±	1.95	3	24.65	±	1.01	4	2.58
490	18.60	±	0.60	4	20.45	±	0.59	3	0.90
493	20.02	±	1.06	3	19.14	±	0.65	4	-0.33
494	20.81	±	1.20	4	22.63	±	0.40	4	0.86
497	21.21	±	1.59	3	22.30	±	0.51	4	0.39
498	19.94	±	1.46	3	20.67	±	1.51	4	0.43

499	18.68 ± 1.32	3	21.96 ± 1.92	3	1.82
500	20.92 ± 0.88	4	22.08 ± 1.04	4	0.54
502	18.27 ± 0.48	3	20.65 ± 0.99	4	1.21
503	19.52 ± 1.82	4	20.04 ± 0.78	4	0.26
504	20.66 ± 1.73	3	23.31 ± 0.16	3	1.34
505	19.27 ± 1.10	4	24.01 ± 0.83	3	2.39
506	20.22 ± 1.41	4	19.15 ± 0.59	4	-0.52
507	19.29 ± 0.95	4	23.23 ± 1.84	4	2.00
514	17.80 ± 0.32	4	18.95 ± 1.39	4	0.63
515	20.51 ± 1.40	4	22.35 ± 1.26	4	0.88
516	19.12 ± 1.41	3	20.19 ± 1.96	4	0.62
520	19.87 ± 0.56	4	22.87 ± 1.45	4	1.48
521	19.45 ± 1.61	4	22.98 ± 1.49	3	1.76
523	20.92 ± 1.19	4	20.63 ± 1.21	4	-0.14
524	18.74 ± 0.70	4	21.05 ± 1.28	4	1.21
525	21.93 ± 0.80	4	20.37 ± 0.76	4	-0.69
527	18.53 ± 1.06	3	21.48 ± 0.99	3	1.43
528	18.92 ± 0.91	3	21.35 ± 1.20	4	1.34
529	22.09 ± 1.51	4	21.40 ± 0.32	4	-0.30
531	19.04 ± 0.82	4	19.52 ± 0.85	4	0.25
532	22.66 ± 1.36	4	24.27 ± 1.83	3	0.63
534	18.20 ± 0.23	4	22.80 ± 0.82	4	2.48
535	16.63 ± 0.34	4	23.49 ± 1.21	4	4.05
536	20.21 ± 0.69	3	22.44 ± 2.03	3	0.94
537	17.99 ± 0.49	4	20.58 ± 1.26	3	1.42
541	19.64 ± 0.33	3	21.11 ± 0.35	3	0.82
543	19.81 ± 1.58	3	20.38 ± 0.66	3	0.15
545	19.01 ± 1.75	3	23.50 ± 1.03	4	2.44
546	20.65 ± 0.70	4	22.02 ± 1.32	4	0.65
548	19.19 ± 1.12	3	23.10 ± 0.86	3	1.94
549	20.47 ± 1.47	4	21.48 ± 1.84	4	0.48
550	19.37 ± 0.81	4	21.33 ± 0.62	4	0.99
552	19.43 ± 0.06	3	21.20 ± 1.02	3	0.81

553	19.46	±	0.87	4	20.36	±	0.76	4	0.46
555	20.32	±	1.46	4	20.70	±	1.42	4	0.18
556	19.85	±	1.67	4	21.48	±	1.73	4	0.80
557	19.17	±	1.60	3	20.64	±	1.10	4	0.69
559	20.92	±	0.64	3	23.32	±	1.52	4	1.06
561	20.08	±	1.48	4	22.03	±	1.96	4	0.95
562	18.23	±	0.54	4	20.71	±	1.61	4	1.33
563	17.60	±	0.56	3	24.12	±	0.82	4	3.79
564	22.04	±	0.74	3	21.42	±	1.32	4	-0.33
565	20.35	±	0.66	4	21.54	±	1.05	3	0.55
570	19.18	±	0.25	4	20.27	±	0.33	4	0.56
571	20.85	±	1.09	4	20.06	±	1.31	4	-0.37
572	20.52	±	1.08	4	20.87	±	0.91	4	0.17
574	19.47	±	0.33	3	21.77	±	0.84	4	1.03
575	18.56	±	0.38	4	21.38	±	1.57	4	1.49
576	18.32	±	0.86	3	22.35	±	1.57	4	2.01
578	19.08	±	0.75	4	21.39	±	1.94	4	1.19
579	19.99	±	1.46	4	20.08	±	0.88	4	0.04
581	19.57	±	2.26	3	22.20	±	1.61	4	1.43
582	17.86	±	0.61	3	20.51	±	3.17	3	1.50
583	20.02	±	1.92	4	20.11	±	0.39	4	0.05
584	17.68	±	0.48	3	20.93	±	0.49	4	1.66
585	18.84	±	0.89	3	20.96	±	1.26	4	1.04
586	20.55	±	2.18	3	20.55	±	1.92	4	0.07
587	20.41	±	0.82	3	20.81	±	1.26	3	0.12
588	18.47	±	0.50	4	22.47	±	0.78	4	2.12
589	21.21	±	1.49	3	20.79	±	0.62	4	-0.13
590	18.22	±	0.44	4	21.07	±	0.28	3	1.63
591	18.96	±	0.76	4	19.94	±	0.54	3	0.49
592	21.10	±	1.22	3	21.96	±	0.82	4	0.50
594	19.74	±	1.92	4	19.91	±	2.73	3	0.09
596	19.21	±	1.46	3	21.71	±	1.46	4	1.21
599	19.77	±	0.70	3	21.27	±	1.59	4	0.85

600	17.69	±	0.35	4	22.72	±	1.44	4	2.79
602	21.66	±	0.72	4	19.63	±	1.37	4	-0.91
603	18.34	±	1.07	3	20.44	±	0.49	4	1.24
604	20.32	±	0.68	3	22.89	±	1.09	4	1.35
605	18.93	±	0.19	3	18.97	±	1.02	3	-0.01
606	19.24	±	1.16	4	23.45	±	1.27	3	2.23
608	20.13	±	0.38	4	22.20	±	1.30	3	0.93
609	19.35	±	1.70	4	24.84	±	1.44	4	2.78
610	20.02	±	1.93	3	20.83	±	0.56	4	0.28
611	20.46	±	0.39	4	21.65	±	1.03	3	0.65
612	17.71	±	0.93	4	21.51	±	1.03	3	2.20
613	19.27	±	0.97	3	20.49	±	1.40	4	0.70
614	18.61	±	1.04	4	22.45	±	1.14	4	2.03
615	20.17	±	1.17	4	24.25	±	0.87	4	1.98
617	19.75	±	1.28	4	22.11	±	0.45	4	1.17
618	18.48	±	0.55	3	21.34	±	1.86	3	1.61
619	20.42	±	1.42	4	20.39	±	1.59	4	-0.01
620	20.61	±	1.33	3	19.44	±	1.14	4	-0.46
622	18.75	±	0.75	4	19.06	±	1.24	3	0.14
623	20.44	±	1.23	4	21.02	±	1.18	4	0.28
624	18.04	±	0.46	4	20.72	±	1.04	4	1.45
625	19.29	±	0.65	3	21.47	±	1.05	4	1.04
626	21.56	±	1.66	4	21.26	±	1.61	4	-0.13
627	20.77	±	1.03	4	20.36	±	0.83	4	-0.19
628	18.84	±	0.90	4	20.04	±	1.42	3	0.60
629	16.97	±	0.46	4	21.42	±	1.53	4	2.57
630	17.36	±	1.08	3	23.07	±	0.87	4	3.13
631	20.03	±	2.02	4	21.58	±	2.05	4	0.76
632	19.34	±	0.95	4	20.83	±	1.09	4	0.75
633	18.33	±	0.63	3	21.08	±	0.29	4	1.40
634	19.40	±	0.54	4	24.00	±	1.54	4	2.32
635	18.07	±	0.31	4	19.70	±	0.78	4	0.88
636	18.90	±	0.77	4	22.24	±	0.63	3	1.65

637	18.68	±	0.92	4	21.35	±	0.68	4	1.40
638	19.29	±	0.78	4	20.72	±	1.21	4	0.73
640	21.12	±	1.25	4	21.75	±	1.09	3	0.22
641	17.98	±	0.81	4	21.07	±	1.43	4	1.69
642	18.49	±	0.42	4	21.10	±	1.03	4	1.39
643	19.97	±	1.52	4	20.63	±	0.66	4	0.32
644	19.37	±	0.67	4	22.19	±	1.97	4	1.42
645	19.89	±	1.96	3	21.05	±	1.58	4	0.65
646	17.87	±	0.93	3	20.96	±	1.55	3	1.93
647	17.71	±	0.70	4	19.45	±	0.90	3	0.94
648	18.60	±	0.35	3	21.87	±	1.66	3	1.68
649	17.85	±	0.98	4	22.48	±	1.16	4	2.55
650	17.66	±	0.78	3	23.36	±	0.95	4	3.27
651	20.47	±	2.29	3	21.78	±	2.32	3	0.58
652	17.18	±	0.43	3	23.84	±	0.36	3	3.62
653	18.22	±	1.03	3	21.13	±	0.79	4	1.69
654	21.50	±	1.52	4	18.52	±	0.50	3	-1.28
655	17.32	±	0.43	4	24.22	±	1.75	3	3.88
656	20.41	±	2.55	4	20.50	±	1.30	4	0.04
657	19.50	±	0.90	3	22.50	±	0.67	4	1.44
658	18.06	±	0.77	3	21.58	±	0.49	3	2.02
659	18.86	±	0.75	4	23.99	±	0.55	3	2.68
660	18.72	±	0.93	4	22.82	±	1.17	4	2.15
661	19.14	±	0.95	4	23.37	±	1.28	4	2.16
662	20.24	±	1.07	4	22.12	±	1.22	4	0.91
663	19.31	±	1.59	4	23.13	±	0.86	4	1.94
665	18.22	±	0.67	4	21.16	±	1.43	3	1.59
667	19.62	±	0.77	4	22.70	±	1.63	4	1.54
668	17.31	±	0.77	3	20.31	±	1.50	4	1.63
670	18.36	±	1.04	4	20.51	±	0.75	4	1.15
671	22.16	±	0.80	4	22.13	±	1.63	4	-0.01
674	20.33	±	2.17	3	21.65	±	0.84	4	0.58
677	20.08	±	1.61	3	22.42	±	1.29	4	1.22

678	18.76	±	0.70	4	20.39	±	1.07	4	0.86
680	19.14	±	1.08	4	19.64	±	1.36	3	0.18
682	19.15	±	0.41	4	21.83	±	1.48	3	1.46
683	17.58	±	0.80	3	23.16	±	1.77	4	3.03
684	18.38	±	0.37	4	20.94	±	1.14	4	1.37
685	19.94	±	1.52	3	19.55	±	1.26	4	-0.12
686	19.98	±	1.31	4	19.79	±	0.78	4	-0.09
687	18.84	±	0.84	3	22.03	±	1.43	4	1.78
688	19.91	±	0.91	3	21.51	±	1.84	3	0.81
690	18.90	±	0.98	3	21.25	±	1.19	4	1.34
58-861	19.80	±	0.65	14	20.93	±	0.46	16	0.56
P-36	19.43	±	0.51	16	21.14	±	0.75	13	0.83
P-64	19.12	±	0.36	16	20.87	±	0.64	16	0.89
Poli	19.64	±	0.61	13	21.68	±	0.70	15	0.95
